# Multiplex CRISPR/Cas9-mediated raffinose synthase gene editing reduces raffinose family oligosaccharides in soybean

**DOI:** 10.3389/fpls.2022.1048967

**Published:** 2022-11-15

**Authors:** Li Cao, Zeru Wang, Hongyu Ma, Tengfei Liu, Jing Ji, Kaixuan Duan

**Affiliations:** Department of Plant Pathology, Nanjing Agricultural University, Nanjing, China

**Keywords:** soybean, CRISPR/Cas9, multiplex gene editing, RS, RFOs

## Abstract

Soybean [*Glycine max* (L.) Merr.] is an important world economic crop. It is rich in oil, protein, and starch, and soluble carbohydrates in soybean seeds are also important for human and livestock consumption. The predominant soluble carbohydrate in soybean seed is composed of sucrose and raffinose family oligosaccharides (RFOs). Among these carbohydrates, only sucrose can be digested by humans and monogastric animals and is beneficial for metabolizable energy, while RFOs are anti-nutritional factors in diets, usually leading to flatulence and indigestion, ultimately reducing energy efficiency. Hence, breeding efforts to remove RFOs from soybean seeds can increase metabolizable energy and improve nutritional quality. The objective of this research is to use the multiplex Clustered Regularly Interspaced Short Palindromic Repeats (CRISPR)/Cas9‐mediated gene editing system to induce the knockout of soybean raffinose synthase (RS) genes *RS2* and *RS3* simultaneously to reduce RFOs in mature seeds. First, we constructed five types of multiplex gene editing systems and compared their editing efficiency in soybean hairy roots. We confirmed that the two-component transcriptional unit (TCTU) and single transcriptional unit (STU) systems with transfer RNA (tRNA) as the cleavage site performed better than other systems. The average editing efficiency at the four targets with TCTU-tRNA and STU-tRNA was 50.5% and 46.7%, respectively. Then, we designed four single-guide RNA (sgRNA) targets to induce mutations at *RS2* and *RS3* by using the TCTU-tRNA system. After the soybean transformation, we obtained several *RS2* and *RS3* mutation plants, and a subset of alleles was successfully transferred to the progeny. We identified null single and double mutants at the T2 generation and analyzed the seed carbohydrate content of their progeny. The *RS2* and *RS3* double mutants and the *RS2* single mutant exhibited dramatically reduced levels of raffinose and stachyose in mature seeds. Further analysis of the growth and development of these mutants showed that there were no penalties on these phenotypes. Our results indicate that knocking out *RS* genes by multiplex CRISPR/Cas9-mediated gene editing is an efficient way to reduce RFOs in soybean. This research demonstrates the potential of using elite soybean cultivars to improve the soybean meal trait by multiplex CRISPR(Clustered Regularly Interspaced Short Palindromic Repeats)/Cas9-mediated gene editing.

## Introduction

Soybean [*Glycine max* (L.) Merr.] cultivars have an average composition of 15% soluble carbohydrates in dry seeds, which are important for human and livestock consumption (Hsu et al., 1973). The predominant soluble carbohydrate component of soybean is composed of sucrose and raffinose family oligosaccharides (RFOs; mainly including raffinose and stachyose) (Openshaw and Hadley, 1978; Hagely et al., 2013). Among these carbohydrates, only sucrose can be digested by humans and monogastric animals and is beneficial for metabolizable energy. RFOs are anti-nutritional factors in diets because they cannot be digested due to the lack of α-galactosidase activity in the gut of humans and monogastric animals. These RFOs are metabolized by anaerobic bacteria present in the gut to release gases such as hydrogen, carbon dioxide, and methane, usually leading to flatulence and indigestion, ultimately reducing energy efficiency (Karr-Lilienthal et al., 2005). Therefore, breeding efforts to remove RFOs from the soybean seed can increase the metabolizable energy of the diet and reduce flatulence production. The biosynthesis pathway of RFOs in plants has been well studied (Sengupta et al., 2015; **Supplementary Figure S1**). Raffinose is synthesized from sucrose and galactinol, catalyzed by raffinose synthases (RSs). Raffinose and galactinol synthesize stachyose with the participation of stachyose synthase (STAS) (Peterbauer and Richter, 2001; Nishizawa et al., 2008). In this pathway, galactinol synthase (GOLS) and RS, which produce galactinol and raffinose, are considered key enzymes in the RFO biosynthesis. As a result, knocking out these genes should lead to increased sucrose and decreased raffinose and stachyose. In soybean, *RS2* and *RS3* are responsible for raffinose synthesis, while GOLSs GmGOLS1A and GmGOLS1B play important roles in galactinol synthesis (Dierking and Bilyeu, 2008; Dierking and Bilyeu, 2009; Hagely et al., 2020; Le et al., 2020). Previous research has been successful in deploying variant alleles of key soybean *RS* genes, leading to reductions in seed RFOs and increases in seed sucrose (Dierking and Bilyeu, 2009; Hagely et al., 2020). RNAi silencing of *RS2* and CRISPR/Cas9-mediated knockout of *GmGOLS1A*/*GmGOLS1B* were also reported to reduce soybean seed RFOs (Valentine et al., 2017; Le et al., 2020). However, the functional redundancy of genes and gene linkage in soybean still makes the selection of the desired traits very difficult.

The CRISPR/Cas9 system has recently become a widely used tool for the generation of mutagenesis and multiplex gene editing for both functional genomics and crop improvement, as it is simple and robust for genome modification. So far, different types of CRISPR/Cas9-mediated multiplex gene editing have been reported in plants. The traditional system is a two-component transcriptional unit (TCTU) in which the Cas9 protein is expressed from an RNA polymerase (Pol) II promoter, whereas the single-guide RNAs (sgRNAs) are typically expressed from separate Pol III promoters, such as the *U6* or *U3* promoter (Lowder et al., 2015; Ma et al., 2015; Zhang et al., 2016; Wang et al., 2018). Alternatively, multiple sgRNAs can be driven by a single Pol III promoter as a single transcript, separated by 20-bp Csy4 hairpins (Tsai et al., 2014) or 77-bp tRNA^Gly^ genes (Xie et al., 2015) as cleavage sites, generating functional individual sgRNAs after processing by Csy4 endoribonuclease or the plant endogenous transfer RNA (tRNA) processing system (Xie et al., 2015; Čermák et al., 2017; Tang et al., 2019). Another system is a single transcriptional unit (STU) in which Cas9 fused with endoribonuclease and multiple sgRNAs is expressed from a single Pol II promoter. An individual expression unit in the whole cassette is separated by Csy4 hairpins or tRNA^Gly^ genes, generating functional individual units after processing (Xie et al., 2015; Čermák et al., 2017). In soybean, the TCTU system was used to increase isoflavone content (Zhang et al., 2020), and STU was used in soybean gene editing (Carrijo et al., 2021). However, the CRISPR/Cas9-mediated multiplex gene editing system for soybean still needs to be evaluated and modified.

In this study, we constructed different types of TCTU and STU systems with different processing mechanisms for soybean and compared their multiplex gene editing efficiency. We subsequently used the highest CRISPR/Cas9 multiplex gene editing system to knock out the soybean *RS2* and *RS3* genes simultaneously. Following the transformation and progeny segregation, we generated stable null alleles at the T2 generation and confirmed that the loss of function of the two genes *RS2* and *RS3* results in the low content of RFOs in soybean seeds. Both single and double mutants showed a significant increase in sucrose and decrease in raffinose and stachyose. Low RFOs were achieved without affecting soybean morphology, including plant height, seed weight, or other agronomic traits. Overall, we successfully produced low-RFO and high-sucrose soybean plants with heritable mutations at multiple genomic loci by CRISPR/Cas9-mediated multiplex gene editing, which has the potential to add value to soybean by improving the metabolizable energy of the meal. Our efforts may provide a novel approach to soybean breeding with low RFOs and high sucrose.

## Materials and methods

### Soybean materials, growth conditions, and morphological characterization

The soybean genotype Williams 82 was used in this study. Soybeans were sown in plastic pots and were grown in a greenhouse under a 16:8-h day and night photoperiod at 28°C. Soybean growth performance, including weight per 100 seeds, pod length, and yield per single plant, was recorded after the soybean reached maturity. Plant height was measured after 1 month of growth.

### Vector construction

The pFGC5941 vector from the Arabidopsis Biological Resource Center (ABRC) was used as a backbone, and the vector map was supplied in **Supplementary Figure S2**. The Cas9 coding sequence driven by the soybean polyubiquitin (*GmUBQ*) promoter was cloned into pFGC5941 by *Eco*R I and *Hind* III to generate pFGC5941-GmUBQ-Cas9. To construct the vector of the TCTU, the sequence including four sgRNAs driven by four individual soybean *GmU6* promoters was synthesized and cloned into pFGC5941-GmUBQ-Cas9 using a recombination method by GenScript (Nanjing, China). For the construction of the TCTU-tRNA, the sequence, including four sgRNAs separated with tRNA^Gly^s, driven by a single *GmU6* promoter, was synthesized and cloned into pFGC5941-GmUBQ-Cas9. To construct the TCTU-Csy4, Cas9 fused with Csy4 by a peptide 2A, which was driven by a *GmUBQ* promoter, was synthesized and cloned into pFGC5941, generating pFGC5941-GmUBQ-Csy4-Cas9. The sequence, including four sgRNAs connected by a Csy4 cleavage site, was synthesized and cloned into pFGC5941-GmUBQ-Csy4-Cas9. For the construction of the STU-tRNA, the sequence, including four sgRNAs separated with tRNA^Gly^s, was synthesized and cloned into pFGC5941-GmUBQ-Cas9 by connecting with another tRNA^Gly^. To construct the STU-Csy4, the sequence, including the four sgRNAs separated by Csy4 cleavage sites, was synthesized and cloned into pFGC5941-GmUBQ-Csy4-Cas9 by connecting with another Csy4 cleavage site. All sequences used here were supplied in the Supplementary Material.

To generate the vector TCTU-tRNA-RS to target *RS2* (Glyma.06G179200) and *RS3* (Glyma.05G003900), the sequence including four sgRNAs targeting *RS2* and *RS3* (**Supplementary Table S1**) separated with tRNAs, which was driven by a single GmU6 promoter, was synthesized and cloned into pFGC5941-GmUBQ-Cas9.

### *Agrobacterium rhizogenes*-mediated soybean hairy root transformation

The method was performed as previously described (Cheng et al., 2021). The *Agrobacterium rhizogenes* strain K599 was used to induce hairy roots. Seeds were surface sterilized for 16 h using chlorine gas produced by mixing 4 ml of 12 N HCl and 100 ml of commercial bleach (5.25% sodium hypochlorite) in a tightly sealed desiccator. The constructed vectors were transformed into *A. rhizogenes* strain K599. Cotyledons from germinated seeds of Williams 82 were infected by agrobacteria. Soybean hairy roots formed 15–20 days after infection and were harvested for genotyping.

### *Agrobacterium*-mediated soybean genetic transformation

The protocol was performed as previously described (Zeng et al., 2004; Paz et al., 2006). The *Agrobacterium tumefaciens* strain AGL1 was used for soybean genetic transformation. Regenerated plants were grown in the greenhouse for seed harvest. Seedlings were screened by herbicide leaf painting with 100 mg/l of glufosinate onto trifoliate leaves. Leaves from herbicide-resistant T0 plants were collected for gene editing analysis.

### Genotyping of transgenic hairy roots and plants

Total genomic DNA was extracted using Hexadecyl trimethyl ammonium bromide(CTAB) method (Dellaporta et al., 1983). Target sequences were amplified from samples by PCR using specific primers (**Supplementary Table S2**). These PCR products were digested with T7 endonuclease I or restriction enzyme *Pst* I. The edited samples were visualized and photographed using the gel imaging system. PCR products from the edited samples were purified and ligated into the TA cloning vector (Takara, Japan) for Sanger sequencing. For transgenic plant identification, leaf painting with 100 mg/l of glufosinate was performed. Gene editing was confirmed by T7EI and Sanger sequencing. All of the PCR conditions were set as follows: one cycle at 95°C for 5 min, followed by 35 cycles at 95°C for 15 s, one cycle at 58°C for 15 s, one cycle at 72°C for 30 s, and a final extension cycle at 72°C for 7 min.

### Analysis of carbohydrate composition in soybean seeds

Soluble carbohydrates in soybean were determined by ultra-performance liquid chromatography-tandem mass spectrometry (UPLC-MS/MS, TQ-S micro, Waters, Milford, MA, USA). The extraction of soluble carbohydrates in soybean seed was performed as described previously (Hagely et al., 2020). The extract was separated on a Waters Acquity BEH Amide high-performance liquid chromatography (HPLC) column (2.1 mm × 150 mm, 1.7 μm) using an acetonitrile–water mixture as the mobile phase. The electrospray ionization tandem quadrupole mass spectrometric analysis was carried out in the negative ion mode using multiple reaction monitoring (MRM). The content of sucrose, raffinose, and stachyose is reported as the percentage of dry seed weight, which can be converted to g kg^-1^.

## Results

### Construction of a multiplex Clustered Regularly Interspaced Short Palindromic Repeats (CRISPR)/Cas9 gene editing system for the soybean

**Figure 1:**
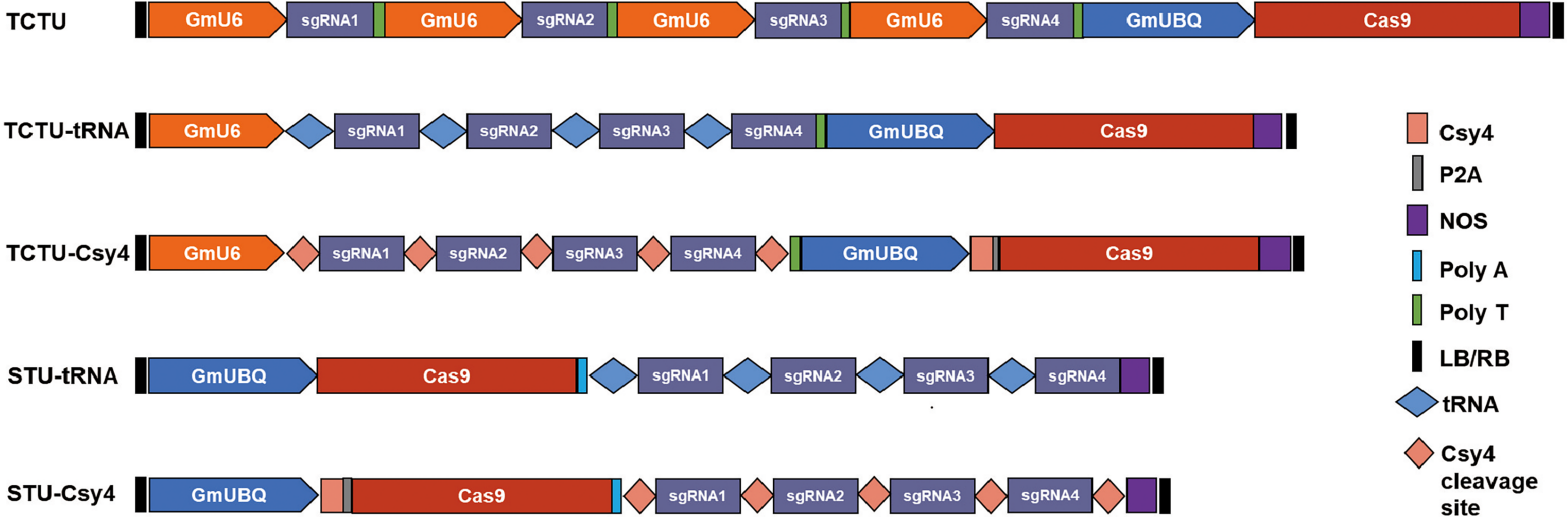
Schematic diagram of the five CRISPR/Cas9 expression systems. TCTU, two-component transcriptional unit system; STU, single transcriptional unit system; *GmUBQ*, *Glycine max* polyubiquitin promoter; NOS, nopaline synthase terminator; *GmU6*, soybean *U6* promoter to express sgRNA; polyT, the terminator of *GmU6*; polyA, a synthetic polyA sequence to facilitate translation; Csy4, RNA endoribonuclease Csy4 (orange rectangle) from *Pseudomonas aeruginosa*; Csy4 cleavage site, a sequence recognized and cleaved by Csy4 (orange diamond); tRNA, 77-bp pre-tRNA^Gly^ gene; P2A, a ribosomal skipping peptide (gray rectangle); and LB/RB, the left and right borders of T-DNA.

**Figure 2:**
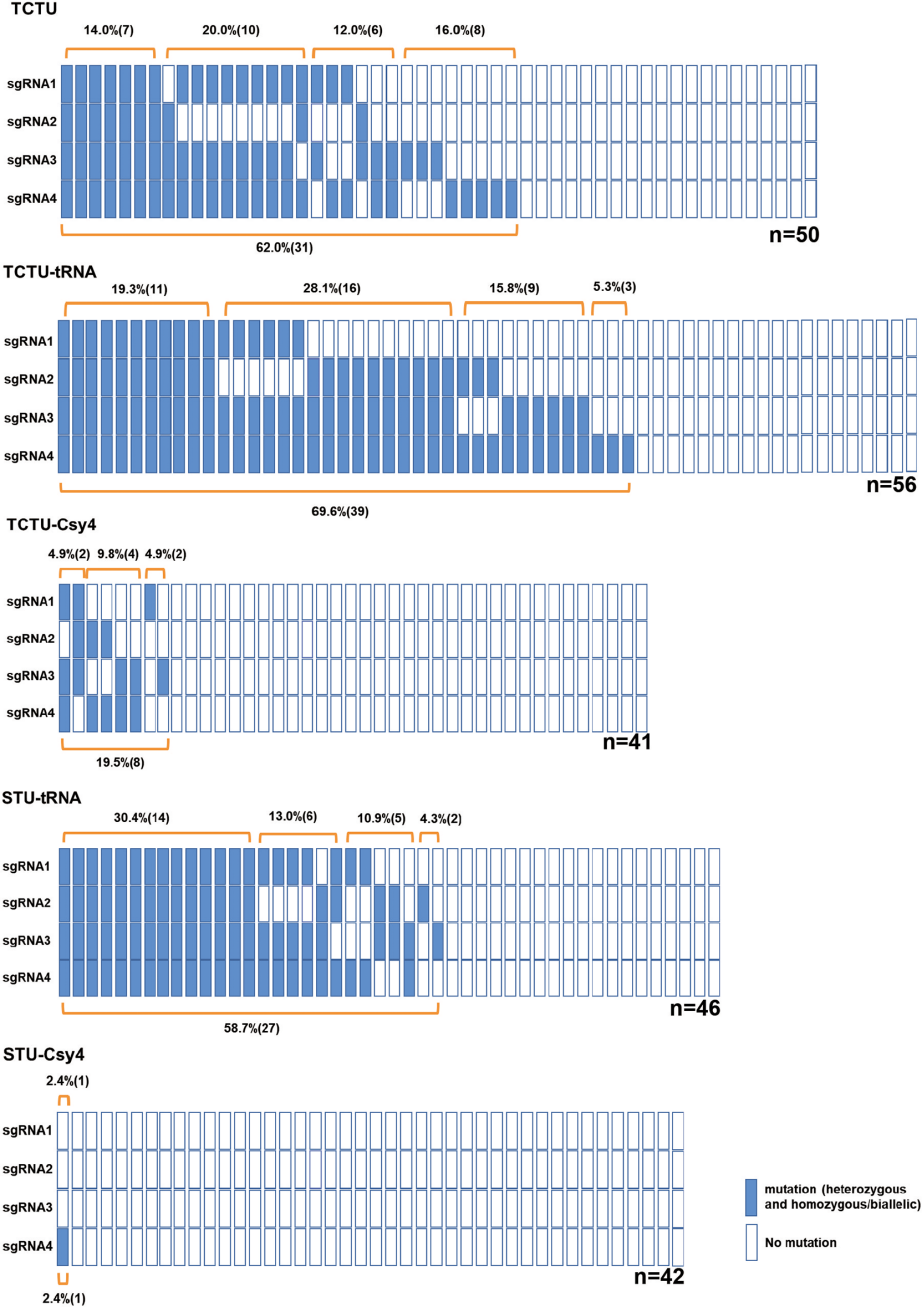
A schematic presentation of the soybean genotyping results for all tested multiplex gene editing systems. Each column (including the four rectangles) represents one tested transgenic event; each rectangle indicates one sgRNA target of this event; the blue rectangles indicate target genes with editing; and the white rectangles indicate the wild type. Mutation efficiency was calculated as the number of mutants divided by the total number of tested events genotyped for each target site (efficiency = editing hairy roots/all hairy roots tested). “n” indicates the number of events tested for each editing system.

To construct and compare various types of multiplex gene editing systems for the soybean, we designed four sgRNAs to target four soybean genes (*Glyma04g37270, Glyma06g17790, Glyma18g216900, *and *Glyma06g14180*) by using the online tool CCTop (Stemmer et al., 2015) (**Supplementary Figure S3**, **Supplementary Table S1**). In the TCTU system, the soybean polyubiquitin promoter (*GmUBQ*) was used to drive the expression of Cas9, and four individual soybean *GmU6* promoters were used to drive the expression of four sgRNAs, respectively. In the TCTU-tRNA system, *GmUBQ* was used to drive Cas9 expression, and four sgRNAs were separated with tRNA^Gly^s, which was driven by a single *GmU6* promoter. In the TCTU-Csy4 system, Csy4 fused with Cas9 by peptide 2A was driven by the *GmUBQ* promoter, and four sgRNAs were connected with the Csy4 cleavage site, driven by a single *GmU6* promoter. In the STU-tRNA system, Cas9 and four sgRNAs were separated with tRNA^Gly^s, driven by a single *GmUBQ* promoter. In the STU-Csy4 system, Csy4 fused with Cas9 by peptide 2A was connected with the four sgRNAs separated by the Csy4 cleavage site, and the whole fused gene was driven by a single *GmUBQ* promoter (**Figure 1**). These constructs were transformed into soybean by *A. rhizogenes*-mediated hairy root transformation. Transgenic hairy roots were harvested and tested to evaluate the multiplex gene editing efficiency of each system. The genotype of the individual event was determined by T7 endonuclease (T7EI) or restriction enzyme assay of the PCR amplicons using the primer set as listed in **Supplementary Table S1**. Noticeably, we define editing efficiency as the sum of roots with evidence of gene editing (which may include the heterozygous, homozygous/biallelic, and chimeric/mosaic mutation types) divided by the total of genotyped roots. Our analysis shows that the TCTU system with tRNA as the cleavage site exhibited the highest editing efficiency, up to 69.6% (efficiency = editing hairy roots/all hairy roots tested). The TCTU system was the second most effective, in which the average editing efficiency was up to 62.0%. The STU system with tRNA as the cleavage site also exhibited a high editing efficiency, up to 58.7% (**Figure 2**; **Supplementary Figures S4**, **S5**). The average editing efficiency of the four genes in the TCTU-tRNA, STU-tRNA, and TCTU systems was up to 50.5%, 46.7%, and 39.0%, respectively (**Table 1**). The system with the Csy4 cleavage site showed the lowest efficiency of most mutation types, particularly in the STU-Csy4 system, in which the editing efficiency was only 2.4% (**Figure 2**, **Table 1**). We further analyzed the effectiveness of these systems for multiplex gene editing at the four targets simultaneously. Consistent with the above result, the TCTU-tRNA, STU-tRNA, and TCTU expression systems performed better in multiplex gene editing. Interestingly, the STU-tRNA system exhibited higher efficiency in editing the four genes simultaneously, up to 30.4%, while the TCTU-tRNA had higher efficiency in editing the three genes simultaneously, up to 28.5%. Systems with the Csy4 cleavage site were the worst in multiple gene editing (**Figure 2**, **Table 1**). Thus, our results confirmed that the systems constructed here are efficient in simultaneously creating multiplexed targeted gene editing using up to four guide RNAs (gRNAs) in soybean, and with tRNA-based expression systems performing best in editing multiple genes simultaneously.

**Table 1:** Editing efficiency induced by various multiplex CRISPR/Cas9 systems for soybean transgenic hairy root.

Construct	Tested events	Targets	Mutation efficiency	Average mutation efficiency	Single mutation efficiency	Double mutation efficiency	Triple mutation efficiency	Quadruple mutation efficiency
**TCTU**	**50**	**sgRNA1**	**38.0% (19)**	**39.0%**	**16.0% (8)**	**12.0% (6)**	**20.0% (10)**	**14.0% (7)**
		**sgRNA2**	**20.0% (10)**					
		**sgRNA3**	**46.0% (23)**					
		**sgRNA4**	**52.0% (26)**					
**TCTU-tRNA**	**56**	**sgRNA1**	**30.4% (17)**	**50.5%**	**5.4% (3)**	**16.1% (9)**	**28.6% (16)**	**19.6% (11)**
		**sgRNA2**	**42.9% (24)**					
		**sgRNA3**	**58.9% (33)**					
		**sgRNA4**	**69.6% (39)**					
**TCTU-Csy4**	**41**	**sgRNA1**	**7.3% (3)**	**9.8%**	**4.9% (2)**	**9.8% (4)**	**4.9% (2)**	**0.0% (0)**
		**sgRNA2**	**9.7% (4)**					
		**sgRNA3**	**9.7% (4)**					
		**sgRNA4**	**12.2% (5)**					
**STU-tRNA**	**46**	**sgRNA1**	**45.6% (21)**	**46.7%**	**4.3% (2)**	**10.9% (5)**	**13.0% (6)**	**30.4% (14)**
		**sgRNA2**	**41.3% (19)**					
		**sgRNA3**	**50.0% (23)**					
		**sgRNA4**	**50.0% (23)**					
**STU-Csy4**	**42**	**sgRNA1**	**0% (0)**	**0.6%**	**2.4% (1)**	**0.0% (0)**	**0.0% (0)**	**0.0% (0)**
		**sgRNA2**	**0% (0)**					
		**sgRNA3**	**0% (0)**					
		**sgRNA4**	**2.4% (1)**					

Mutation was defined as samples with gene editing after the T7EI assay. Mutation efficiency was calculated as the number of mutants divided by the total number of tested events genotyped for each target site. Average mutation means the average mutation of the four sgRNAs. The numbers in brackets mean the number of mutated samples.

### Generation of *RS* mutations in T0 transgenic soybean by multiplex CRISPR/Cas9 gene editing system

**Figure 3:**
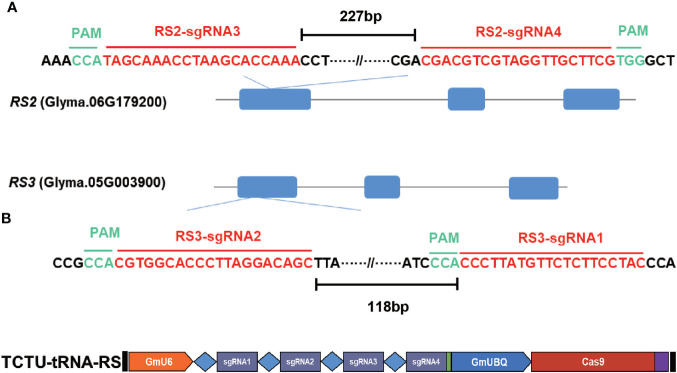
Soybean *RS2* and *RS3* gene structure and target sequence locations. **(A)** Target sequence of *RS2* and *RS3*; the guide sequence is marked in red, and the PAM motif (NGG) is highlighted in green. The distance values between the two sgRNAs were 227 and 118 bp, respectively. **(B)** The schematic diagram of TCTU-tRNA-RS vector targeting *RS2* and *RS3*.

**Figure 4:**
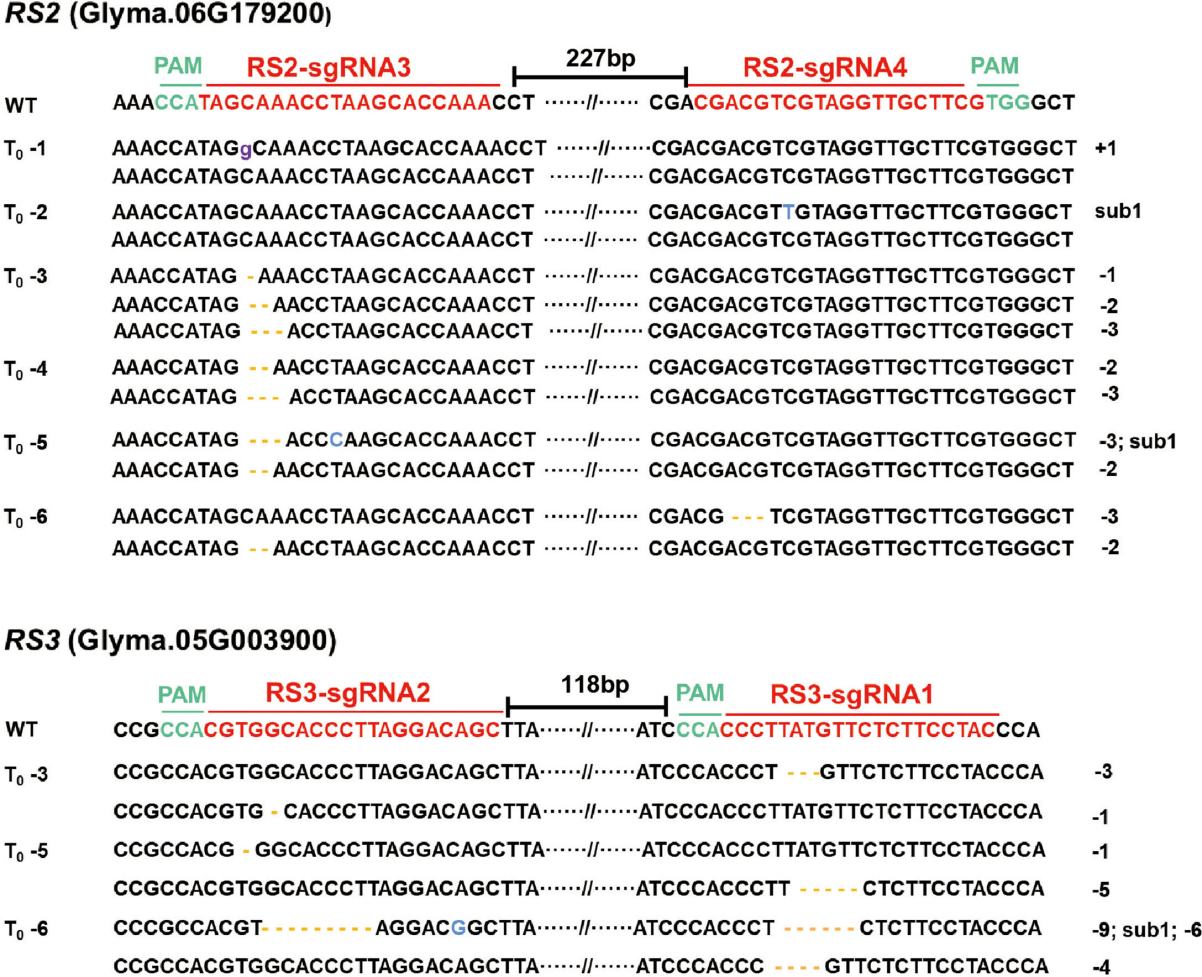
Genotypes of the targeted regions in the *RS2* and *RS3* genes in soybean T0 transgenic plants. Targeted sequences (sgRNAs) are indicated by the red color, and the PAM sequences are highlighted in green. The distance values between the two sgRNAs were 227 and 118 bp, respectively. The base insertion is marked in purple; the base substitution is labeled in blue; and the base deletion is highlighted in yellow bars. “-” means deletion; “+” means insertion; and “sub1” means one base was substituted.

To generate the *RS2* and *RS3* soybean null mutants, we used the TCTU-tRNA system to knock out the *RS2* and *RS3* genes simultaneously. We designed four specific sgRNAs to target these two genes by using the online tool CCTop (**Supplementary Table S1**). These target sequences are located within exon 1 of both *RS* genes (**Figure 3A**). According to the method described above, the vector of TCTU-tRNA-RS, targeting both *RS2* and *RS3* simultaneously, was constructed (**Figure 3B**). To validate the efficiency of this vector in inducing target mutations, we first transferred this plasmid into *A. rhizogenes* strain K599 for hairy root transformation. After infecting the soybean, we tested the gene editing efficiency of these four targets in transgenic hairy roots. The T7EI assay results showed that these four sgRNAs were all effective in inducing *RS2* and *RS3* gene mutation, with the efficiency ranging from 25.8% to 71.0%, and the efficiency of editing four genes simultaneously was up to 12.9% (**Table 2**, **Supplementary Figure S6**), demonstrating the high efficiency of our construct for the next soybean genetic transformation. Following the *Agrobacterium*-mediated genetic transformation using soybean cotyledonary nodes, we successfully obtained six independent soybean T0 transgenic plants screened by herbicide leaf painting (**Supplementary Figure S7**). We subsequently amplified the DNA sequences spanning the targeted regions in *RS2* and *RS3* from the T0 transgenic plants. After the T7EI assay and Sanger sequencing of the cloned PCR products, we found that these four sgRNAs were all effective in inducing *RS2* and *RS3* gene mutations in transgenic plants, and sgRNA3 worked best. There were five transgenic lines mutated at the sgRNA3 target (**Table 2**, **Supplementary Figure S8**). Different transgenic lines had different editing outcomes and harbored several missense mutation types independently, including one-base insertion, one-base substitution, or one-, two-, three-, four-, five-, six-, or nine-base deletions. Surprisingly, line 3 carried chimeric mutations at *RS2* (**Figure 4**, **Supplementary Figure S9**). All of these results indicated the high efficiency of our designed CRISPR/Cas9 construct for soybean multiplex gene editing, and several independent *RS* edited lines were successfully obtained.

**Table 2:** Editing efficiency of the *RS2* and *RS3* targets in transformed events induced by the multiplex CRISPR/Cas9 system TCTU-tRNA-RS.

Transformation type	Tested events	Targets	Mutation frequency	Average mutation	Single mutation frequency	Double mutation frequency	Triple mutation frequency	Quadruple mutation frequency
**Hairy root**	**31**	**sgRNA1**	**41.9% (13)**	**49.2%**	**6.4% (2)**	**22.6% (7)**	**25.8% (8)**	**12.9% (4)**
		**sgRNA2**	**71.0% (22)**					
		**sgRNA3**	**58.1% (18)**					
		**sgRNA4**	**25.8% (8)**					
**Cotyledon**	**6**	**sgRNA1**	**50.0% (3)**	**54.2%**	**50.0% (3)**	**0% (0)**	**33.3% (2)**	**16.7% (1)**
		**sgRNA2**	**50.0% (3)**					
		**sgRNA3**	**83.3% (5)**					
		**sgRNA4**	**33.3% (2)**					

Hairy root means that the editing efficiency of the RS2 and RS3 targets were tested in a soybean transgenic hairy system. Cotyledon means that the editing efficiency of the RS2 and RS3 targets were tested in soybean T0 transgenic plants. The numbers in brackets mean the number of mutated samples.

### Inheritance and segregation of *RS2* and *RS3* mutants

**Figure 5:**
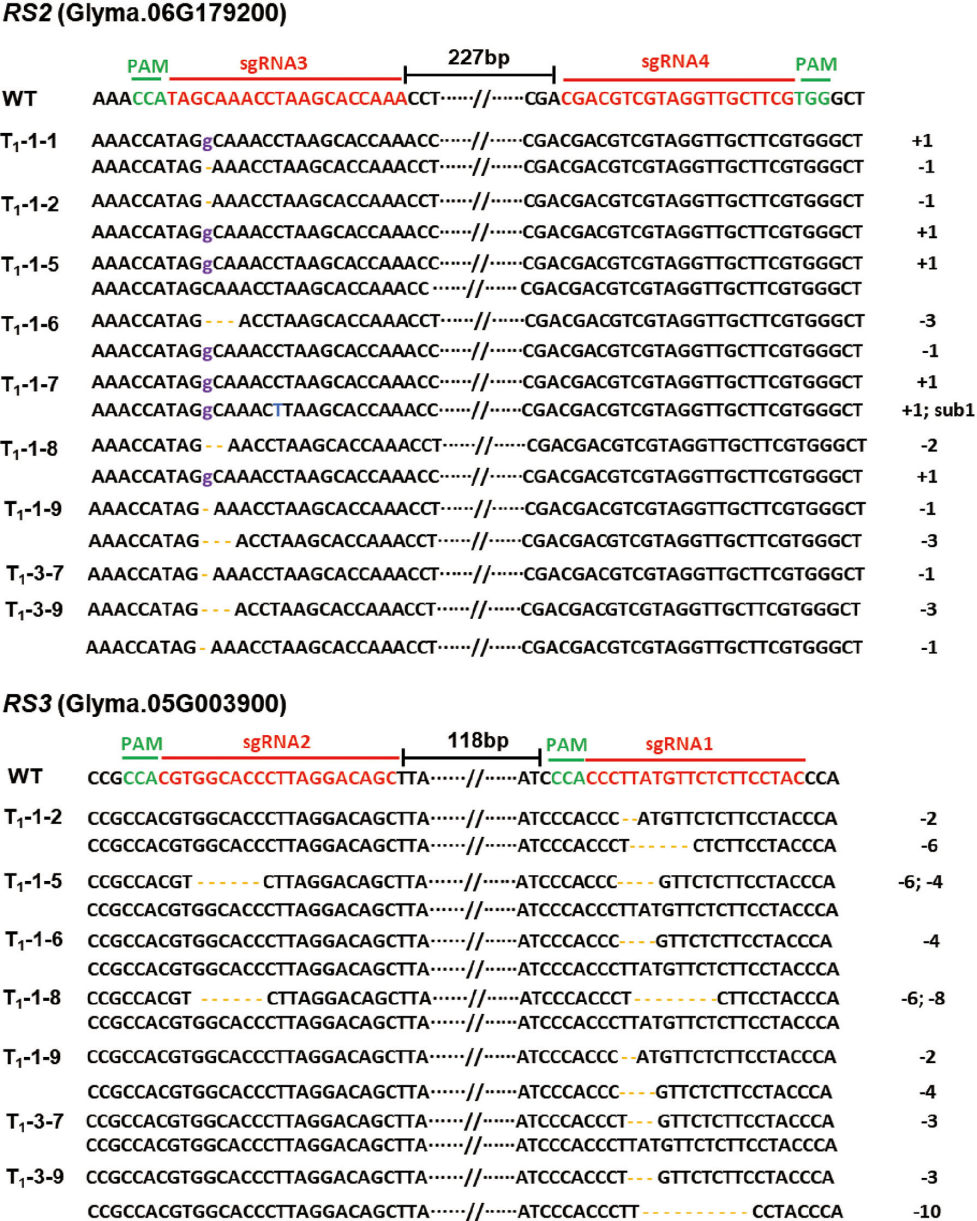
Inheritance and segregation of the *RS2* and *RS3* mutations in T1 transgenic plants. Targeted sequences (sgRNAs) are indicated by the red color, and the PAM sequences are highlighted in green. The distance values between the two sgRNAs were 227 and 118 bp, respectively. The base insertion is marked in purple; the base substitution is labeled in blue; and the base deletion is highlighted in yellow bars. “-” means deletion; “+” means insertion; and “sub1” means one base was substituted.

**Figure 6:**
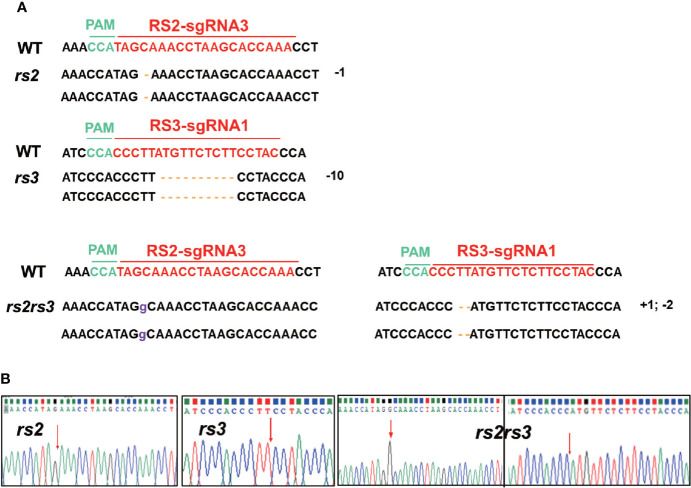
Selected homozygous double and single mutants of *RS2* and *RS3* in T2 transgenic plants. **(A)** Genotypes of selected *RS2* and *RS3* double and single mutants. Targeted sequences (sgRNAs) are indicated by the red color, and the PAM sequences are highlighted in green. The base insertion is marked in purple, and the base deletion is highlighted in yellow bars. “-” means deletion and “+” means insertion. **(B)** Sanger sequencing of *RS2* and *RS3* gene in double or single mutants. The arrow indicates the mutation location.

To identify the stable inheritance of *RS2* and *RS3* mutations in progeny after self-cross, we sowed T1 seeds in the greenhouse and then screened them by herbicide leaf painting. We successfully identified the segregation and inheritable lines of *RS2* and *RS3* mutants in the T1 generation. After Sanger sequencing of PCR clones from the *RS2* and *RS3* mutants, the mutation types of different lines were confirmed. The results showed that missense mutations of one-base insertion, one, two-base deletions in *RS2*, and four-base deletion in *RS3* were heritable successfully. Interestingly, a new missense mutation, a 10-base deletion, occurred in *RS3* (**Figure 5**). However, from our results, we did not find homozygous mutants in both *RS2* and *RS3*. Most *RS2* and *RS3* mutant lines were biallelic or heterozygous (**Figure 5**), so homozygous mutant alleles required further screening from the progeny of the T2 generation. The mutation of *RS2* and *RS3* in T2 plants was also identified by Sanger sequencing. The stable inheritance of all induced mutant alleles of both *RS2* and *RS3* genes was confirmed at the T2 generation. After screening by PCR product sequencing, we finally selected three independent edited lines from T2 progenies, which were Cas9 null segregants and homozygous with a 1-bp deletion at *RS2* for *rs2*, 10-bp deletion at *RS3* for *rs3*, 1-bp insertion at *RS2*, and 2-bp deletion at *RS3* for *rs2rs3* (**Figure 6**). Then, T3 generation seeds from these lines were used for subsequent phenotypic characterizations.

### Carbohydrate composition of *RS* mutants in soybean seeds

*RS2* and *RS3* have been identified and associated with the high-sucrose and low-RFO phenotype by a forward genetic and RNAi screening (Dierking and Bilyeu, 2009; Bilyeu and Wiebold, 2016; Valentine et al., 2017; Hagely et al., 2020). Therefore, we thought that knocking out *RS* genes simultaneously by multiplex CRISPR/Cas9 in soybean would affect the final sucrose and RFO composition in mature seeds. To evaluate the seed carbohydrate content of *RS* mutants, soluble carbohydrates in mutant mature seeds were extracted and detected by UPLC. In our results, the wild type (WT; Williams 82) and *RS2 RS3* null mutant had a significant difference in seed sucrose, raffinose, and stachyose content. *rs2* and *rs2rs3* had significantly more sucrose than WT and *rs3*, with the average percentages being 10.2%, 8.7%, 7.7%, and 7.9%, respectively. Particularly in the *rs2rs3* double mutant, the sucrose content was the highest in all the lines tested (**Table 3**). The raffinose content in *rs2* and *rs2rs3* mutants was significantly lower than that in WT and *rs3*, with average percentages at 0.3%, 0.2%, 0.9%, and 1.0%, respectively (**Table 3**). Stachyose in WT and *rs3* mutants was also significantly higher than that in *rs2* and *rs2rs3* lines, with average percentages of 4.6%, 4.4%, 3.8%, and 2.9%, respectively (**Table 3**). In these lines, the *rs2rs3* double mutant exhibited the highest sucrose content and the least raffinose and stachyose contents. Based on these results, lines with *RS2* mutation had a low RFO and higher sucrose phenotype, while lines with *RS2 RS3* mutation combinations exhibited the lowest RFO and the highest sucrose phenotype.

**Table 3:** Genotype and seed carbohydrate composition for soybean lines with the *RS2* and *RS3* mutation.

Genotype	Sucrose (%)	Raffinose (%)	Stachyose (%)
**WT (Williams 82)**	**7.7 ± 0.2 a**	**1.0 ± 0.1 a**	**4.6 ± 0.2 a**
***rs2***	**8.7 ± 0.4 b**	**0.3 ± 0.1 b**	**3.8 ± 0.4 b**
***rs3***	**7.9 ± 0.2 a**	**0.9 ± 0.1 a**	**4.4 ± 0.3 a**
***rs2rs3***	**10.2 ± 0.6 b**	**0.2 ± 0.1 b**	**2.9 ± 0.3 c**

Carbohydrate composition is expressed as % seed weight. Within a column, means followed by the same letter were not significantly different from each other using the Student’s t-test analysis, P < 0.05.

### *RS* mutant growth and morphology analysis

**Figure 7:**
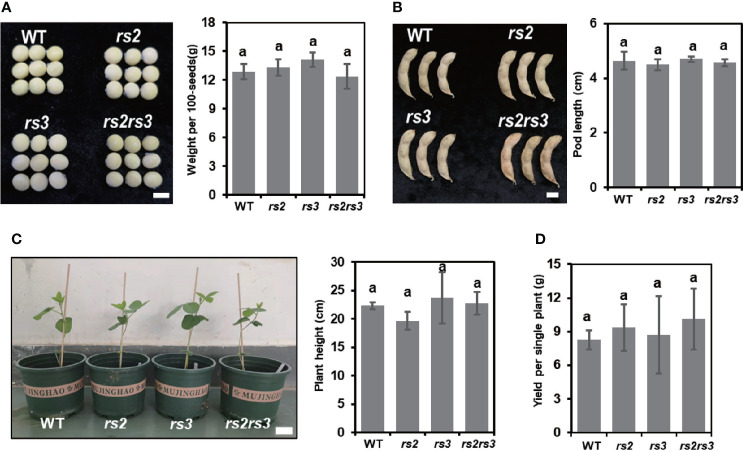
Growth and development phenotype of the *RS2* and *RS3* mutants. **(A)** Comparison of weight per 100 seeds in wild type and null mutants. The bar indicates 0.5 cm. **(B)** Pod length of the wild type and *RS2* and *RS3* null mutant lines. The bar indicates 1 cm. **(C)** Comparison of plant height in wild type and null mutants after 1-month of growth in the greenhouse. The bar indicates 5 cm. **(D)** Comparison of grain yield per single plant in wild type and null mutants. Means followed by the same letter were not significantly different from each other using the Student’s *t*-test analysis, P < 0.05.

To evaluate whether there are growth and development penalties due to the *RS2* and *RS3* mutations in soybean, homozygous T3 soybean mutants were grown under greenhouse conditions for morphology analysis. We compared the plant performance between WT and *RS* null mutants. No visible differences in plant growth or maturation were observed. We observed no significant differences in plant height and pod length. Furthermore, the tested *rs* mutants showed no change in seed weight and yield per single plant as compared to WT plants (**Figure 7**). Altogether, these results indicate that knocking out *RS2* and *RS3* had no effect on soybean growth and morphology under greenhouse conditions.

## Discussion

CRISPR/Cas9-mediated gene editing has been used in the generation of mutagenesis since its discovery, and new advances have made this system even more efficient, especially in multiplex gene editing. Now, several strategies have been used to express multiple gRNAs in multiplex gene editing. First, multiple gRNAs can be produced by the tandem expression of Pol III promoter-driven expression cassettes, namely, the TCTU system (Cong et al., 2013). Second, multiple gRNAs can be transcribed into a single transcript from a Pol II or Pol III promoter, and mature gRNAs can then be released by tRNA processing, ribozyme self-cleavage, or Csy4 ribonuclease cleavage, namely, the STU system (Gao, 2021). For soybean, some reports have shown successful gene editing after stable transformation (Li et al., 2015; Cai et al., 2018; Curtin et al., 2018). However, both editing and editing inheritance were found to be low in these reports. In soybean multiplex gene editing, previous work reported the application of four sgRNAs with two different soybean Pol III-dependent promoters (*GmU3* and *GmU6*) driving the expression of the individual sgRNAs, and this system was used to knock out flavanone-3-hydroxylase (F3H) and flavone synthase II (FNS II) gene to increase the isoflavone content (Zhang et al., 2020). The TCTU and STU systems with three targets were evaluated in soybean hairy roots (Carrijo et al., 2021). They found that the TCTU was more efficient than the STU system. Considering the complex genome of soybean, more efficient strategies for multiplex gene editing are of great interest, mainly for breeding programs. In this work, we developed and evaluated the TCTU and STU multiplex CRISPR/Cas9 systems with tRNA^Gly^ or Csy4 processing machinery that allows four or more sgRNAs to work simultaneously in soybean. We designed four sgRNAs targeting four genes. Our results showed that the TCTU and STU were effective in editing soybean genes, but the efficiency had significant differences between each other. In the TCTU system with soybean Pol III promoter (*GmU6*) driving the expression of the individual sgRNAs, the percentage of editing roots was up to 62.0%, and the percentage of editing roots with four-gene editing simultaneously was up to 14.0% (**Figure 2**, **Table 1**). The TCTU-tRNA system, relying on the plant endogenous tRNA processing machinery to cleave the sgRNAs, was best in editing efficiency, up to 69.6%. The efficiency of editing four genes simultaneously was up to 19.3% (**Figure 2**, **Table 1**). The result of genetic transformation in soybean also showed TCTU-tRNA performing well in multiplex gene editing (**Table 2**). In the STU-tRNA system, with the expression of all sgRNAs and Cas9 driven by the *GmUBQ* promoter, the editing efficiency was up to 58.7%, and the efficiency of editing four genes simultaneously was the highest in all systems, up to 30.4% (**Figure 2**, **Table 1**). While in the TCTU-Csy4 system, relying on Csy4 RNA cleaving, the editing efficiency was much lower, only 19.5%. The STU-Csy4 system was the worst editing system tested here, and only in one sample with a mutation at target 4 (**Figure 2**, **Table 1**). From these results, we summarize that editing systems with tRNA as the cleavage site exhibited better performance in multiplex gene editing, and the TCTU system was also effective in multiple gene editing. However, systems relying on Csy4 RNA cleaving machinery exhibited low editing efficiency, especially in the STU-Csy4. This result is consistent with that of previous reports (Tang et al., 2019; Huang et al., 2020; Huang et al., 2022). We think that this may be due to the expression level of Csy4 in our system. Here, we used peptide 2A to fuse Csy4 and Cas9, but we did not detect the protein level of Csy4, and the individual Csy4 protein may not be enough for the effective cleavage of sgRNAs. Regardless, the technologies described here will be the foundation for efficiently editing multiple genes in the soybean genome. Another phenomenon is the mosaicism of gene editing in transgenic plants. The mutation types may include the heterozygous, homozygous/biallelic, and chimeric/mosaic in transgenic hairy roots. This may be attributed to the possibility that Cas9 is active at different time points or cells during the soybean transformation process, although a constitutive promoter was used to drive its expression.

Soluble carbohydrates in soybean are contributors to human and livestock metabolizable energy. However, in the soybean carbohydrate profiles, RFOs are predominantly indigestible carbohydrates, usually causing the feed to pass quickly through the digestive system, reducing the amount of feed energy (Coon et al., 1990). RFOs are metabolized by anaerobic bacteria present in the gut to release gases such as hydrogen, carbon dioxide, and methane, leading to flatulence and indigestion (Karr-Lilienthal et al., 2005). Previous soybean breeding efforts had identified two major mutations in the soybean gene, *RS2* and *RS3*, associated with the low-RFO phenotype, and the mutation at *RS2* and *RS3* simultaneously resulted in an ultra-low RFO content in soybean (Dierking and Bilyeu, 2009; Bilyeu and Wiebold, 2016; Hagely et al., 2020). RNAi-mediated silencing of the *RS2* gene resulted in a low-RFO phenotype in soybean (Valentine et al., 2017). CRISPR/Cas9-mediated knockout of GOLS genes *GmGOLS1A*/*GmGOLS1B* also reduced RFOs in soybean seeds (Le et al., 2020). In this study, we used the multiplex CRISPR/Cas9 gene editing system to knock out the soybean *RS2* and *RS3* genes simultaneously. We successfully obtained several *RS* null mutants. All of these mutants showed relatively variable sucrose, raffinose, and stachyose contents. Low-RFO and high-sucrose phenotypes were expected in transgenic plants without growth and development penalties. There is a potential use of the CRISPR/Cas9 system for the improvement of other elite soybean cultivars. Considering the time-consuming introgression of the desired *RS2* and *RS3* alleles to elite cultivars, breeding by CRISPR/Cas9 will be faster. From the double mutant, we found that there were still some RFOs left, especially stachyose. Because of the paleopolyploidy of soybean, there may be other genes associated with RFO synthesis aside from *RS2* and *RS3*. Hence, further studies are needed to find new ways to eliminate all RFOs in the soybean. More genes in the RFO synthesis pathway such as *GOLS* and *STAS* can be edited simultaneously in future work.

## Conclusion

In summary, we constructed various types of multiplex gene editing systems for soybean and evaluated their editing efficiency. We found that the TCTU and STU systems with tRNA as the cleavage site had better editing efficiency. The TCTU system with the soybean Pol III promoter (*GmU6*) driving the expression of the individual sgRNAs was also efficient. By using the robust gene editing system, we obtained several *RS* null mutants of the soybean. We confirmed that the loss of function of two the genes, *RS2* and *RS3*, results in the low content of RFOs in soybean. We successfully produced soybean plants with low RFOs and high sucrose by multiplex gene editing, which has the potential to improve the metabolizable energy of the soybean meal.

## Data availability statement

The original contributions presented in the study are included in the article/**Supplementary Material**. Further inquiries can be directed to the corresponding author.

## Author contributions

KD designed the experiments; LC, ZW, TL, and JJ performed the experiments; and HM helped in carbohydrate detection. KD wrote the manuscript. All authors read and approved the final manuscript.

## Funding

This research was supported by grants from the National Science Foundation of China (NSFC; 32172499, 31901957).

## Conflict of interest

The authors declare that the research was conducted in the absence of any commercial or financial relationships that could be construed as a potential conflict of interest.

## Publisher’s note

All claims expressed in this article are solely those of the authors and do not necessarily represent those of their affiliated organizations, or those of the publisher, the editors and the reviewers. Any product that may be evaluated in this article, or claim that may be made by its manufacturer, is not guaranteed or endorsed by the publisher.
