# Supplementary material for: Multiplex CRISPR/Cas9-mediated raffinose synthase gene editing reduces raffinose family oligosaccharides in soybean
Source: Front Plant Sci. 2022 Nov 15;13:1048967. doi: 10.3389/fpls.2022.1048967 (PMC9706108; doi:10.3389/fpls.2022.1048967)
Supplement: Supplementary file 1 [file DataSheet_1.docx]

**
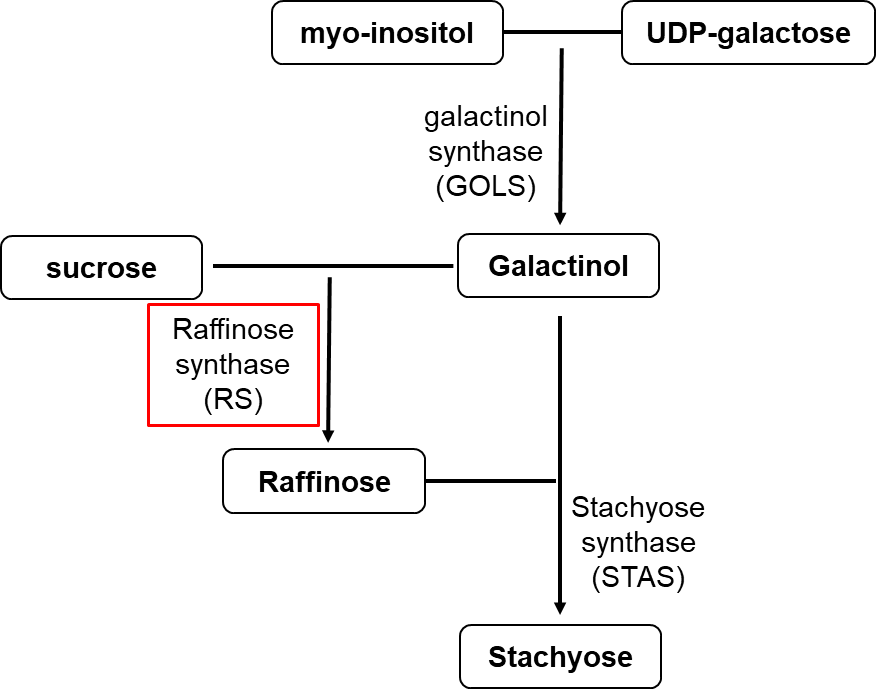
**

**Supplementary Figure 1.** The RFO biosynthesis pathway in plant.


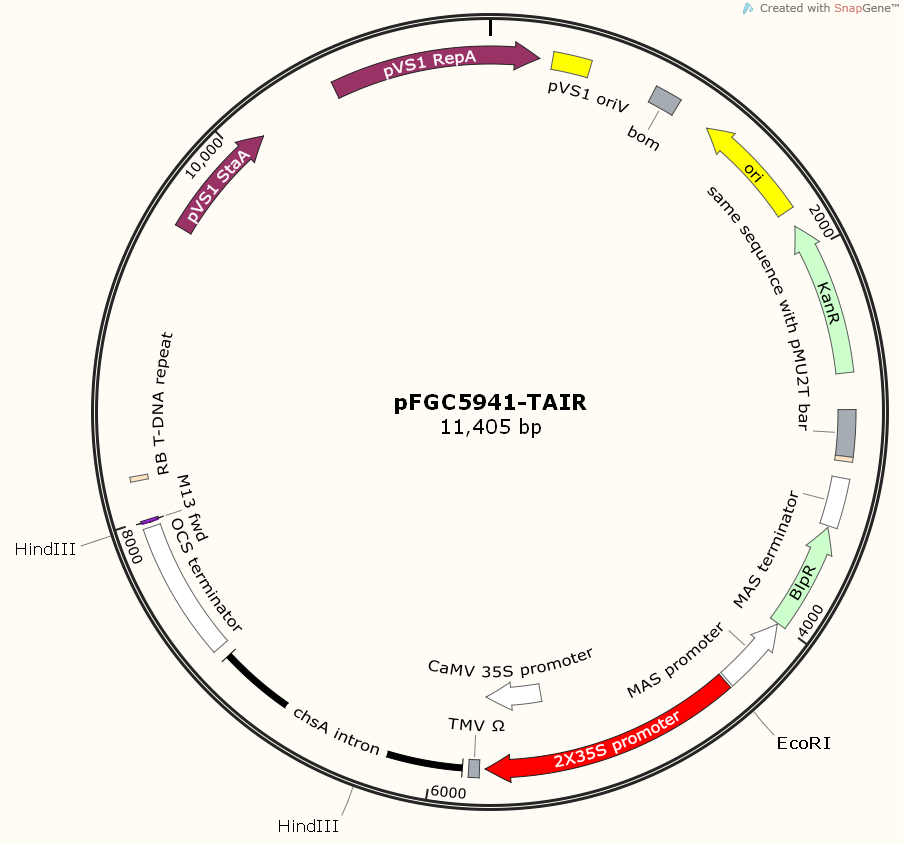


**Supplementary Figure 2.** The map of pFGC5941.

**
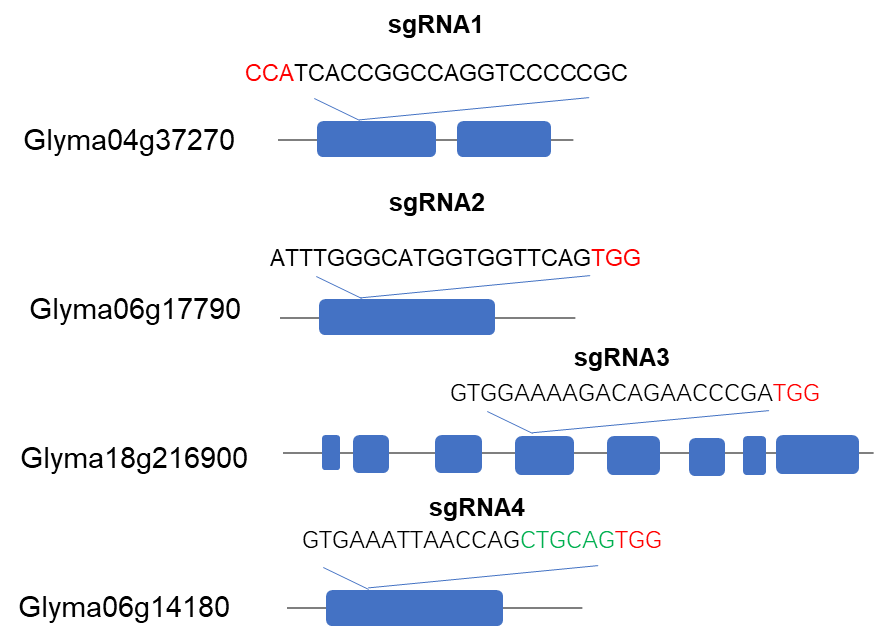
**

**Supplementary Figure 3.** The target sequence locations of selected four soybean genes. PAM was labeled by red color and *Pst* I site was labeled by green color.

**
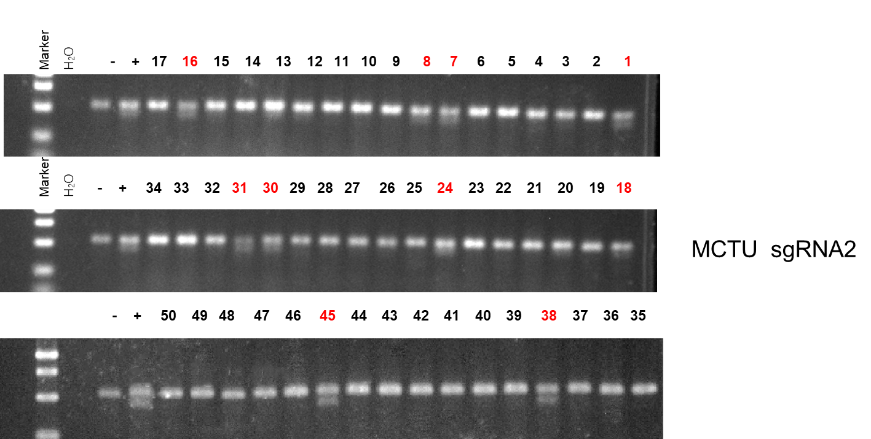

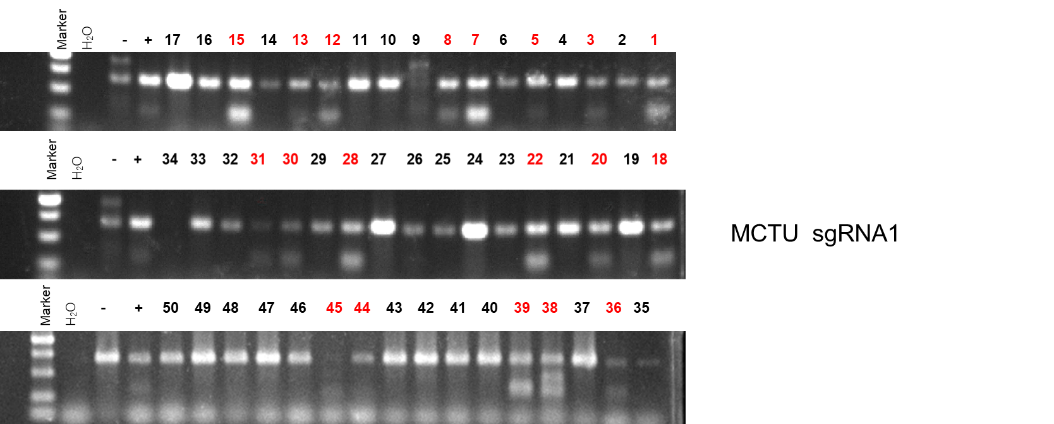
TCTU-sgRNA1 TCTU-sgRNA2**

**
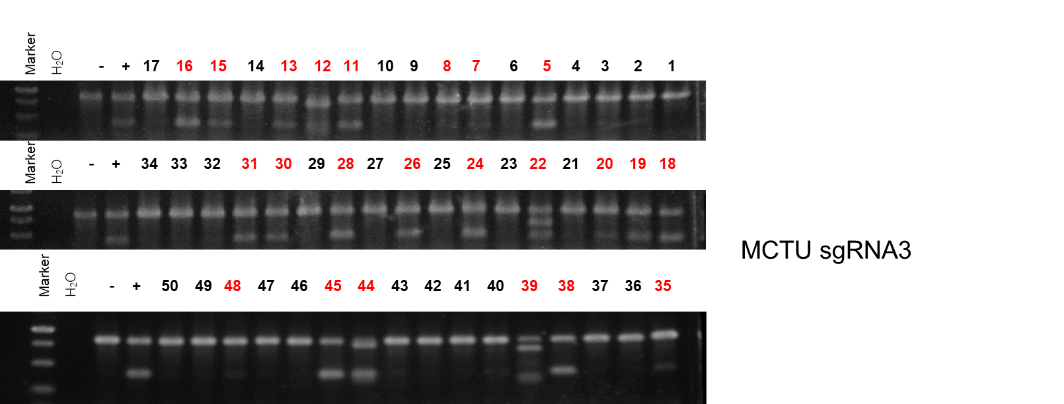
TCTU-sgRNA3 TCTU-sgRNA4**


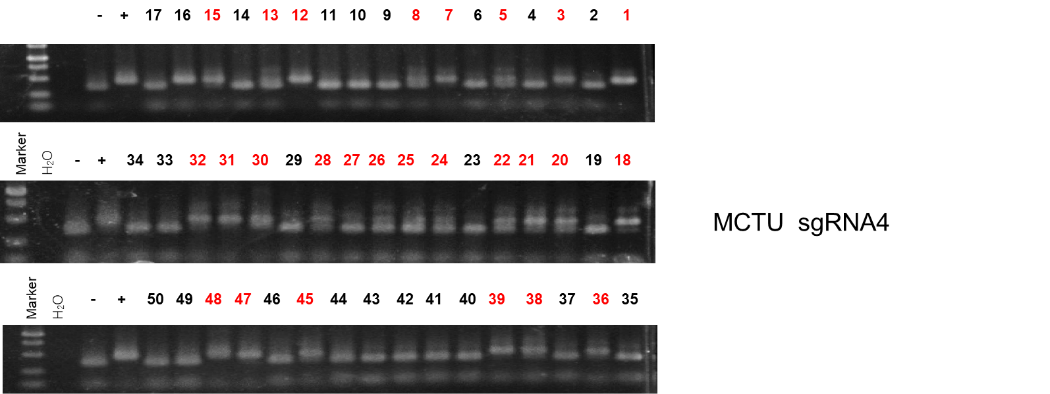


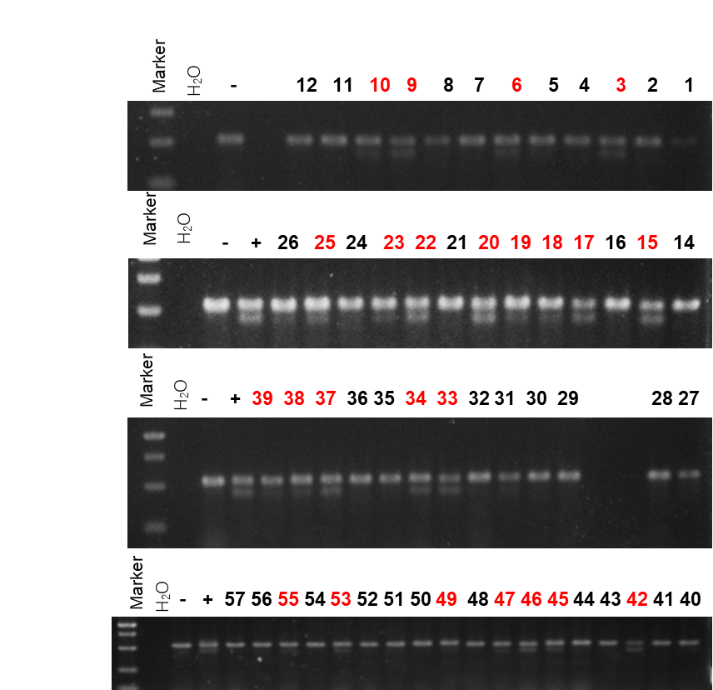
**
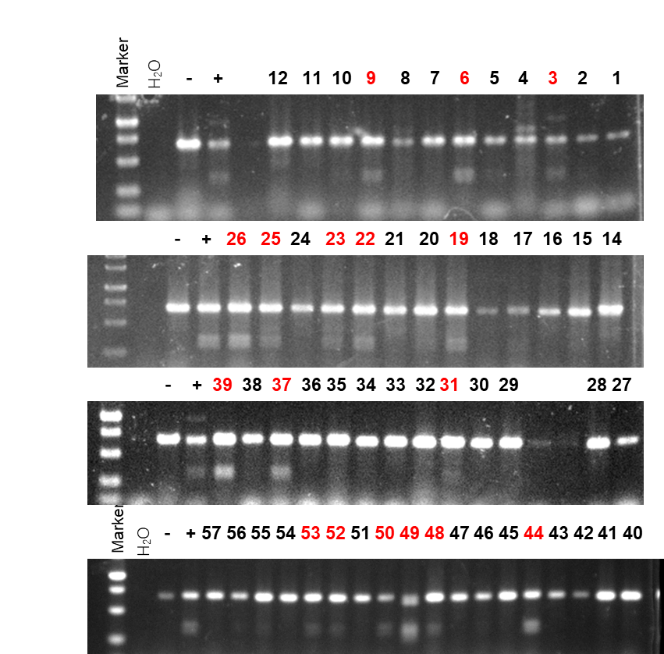
TCTU-tRNA-sgRNA1 TCTU-tRNA-sgRNA2**

Continued

**
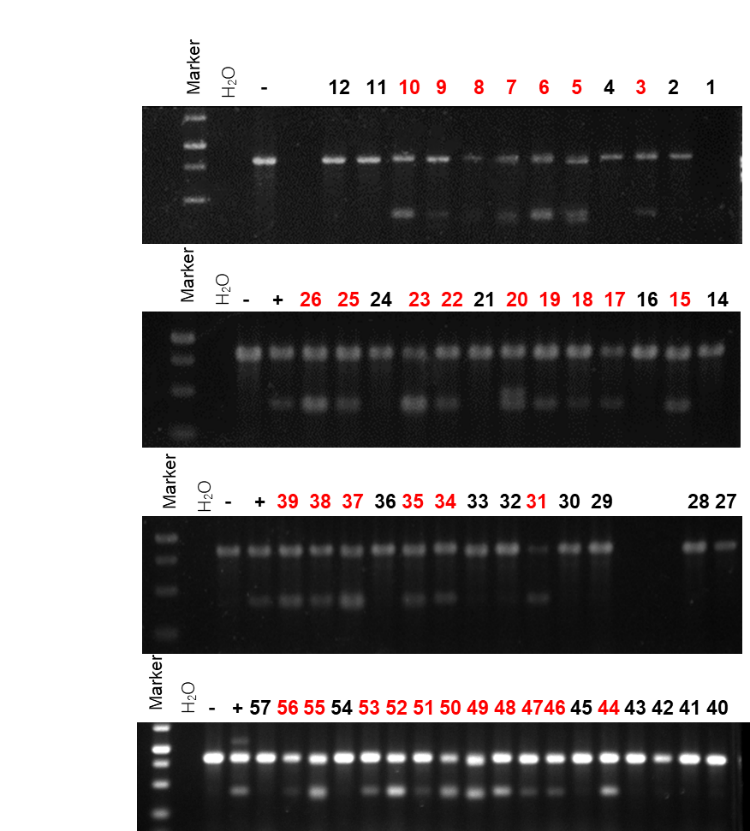
TCTU-tRNA-sgRNA3 TCTU-tRNA-sgRNA4**


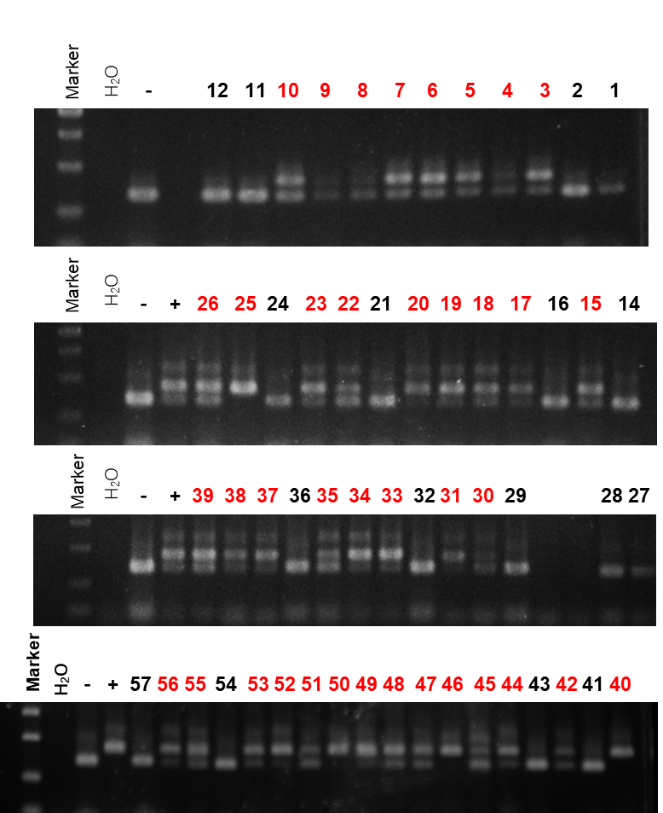


**
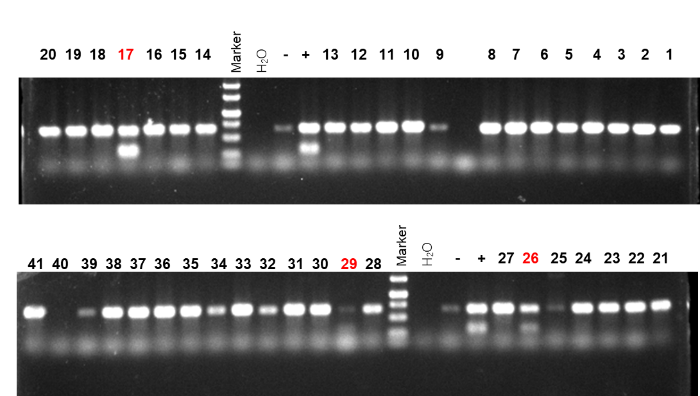
TCTU-Csy4-sgRNA1 TCTU-Csy4-sgRNA2**


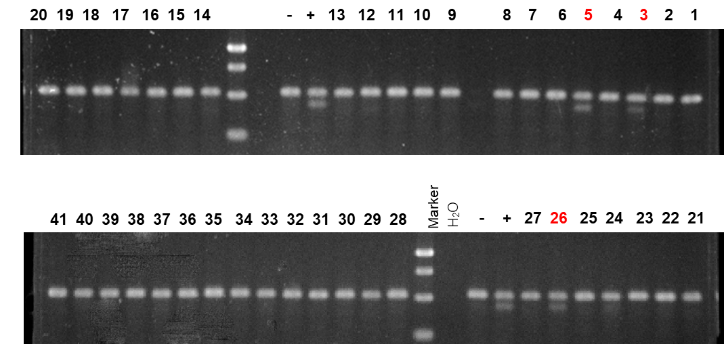


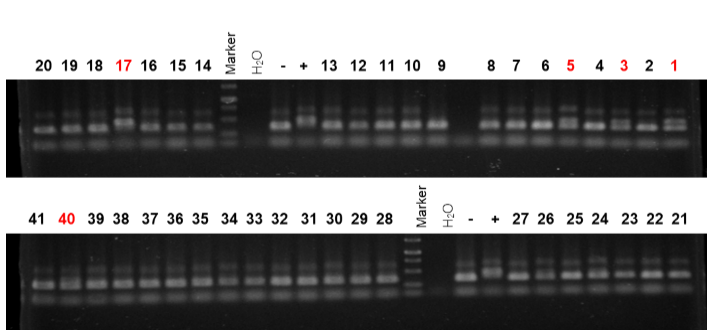
**TCTU-Csy4-sgRNA3 TCTU-Csy4-sgRNA4**

**
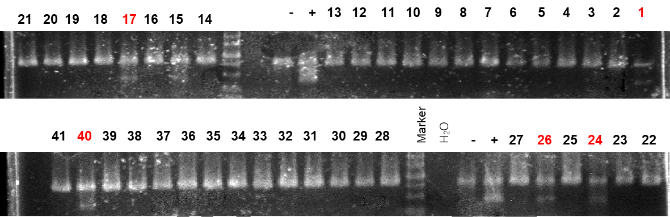
**

Continued

**STU-tRNA-sgRNA1 STU-tRNA-sgRNA2**


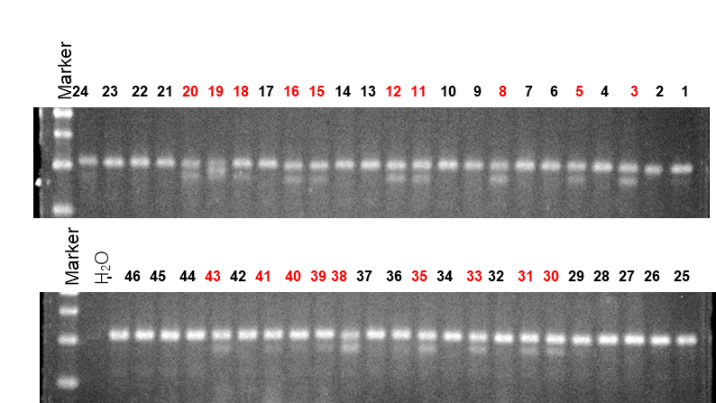

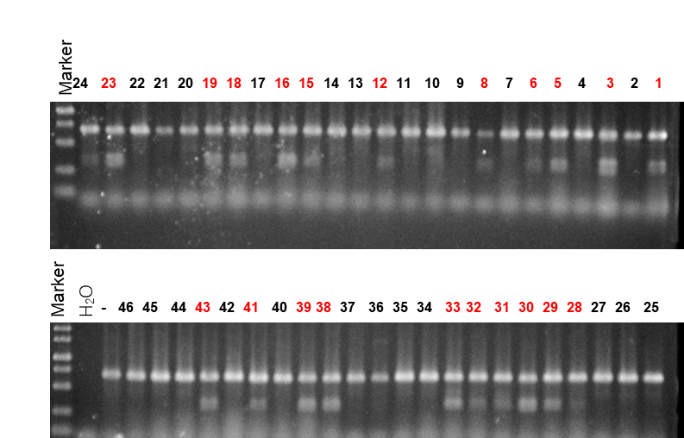


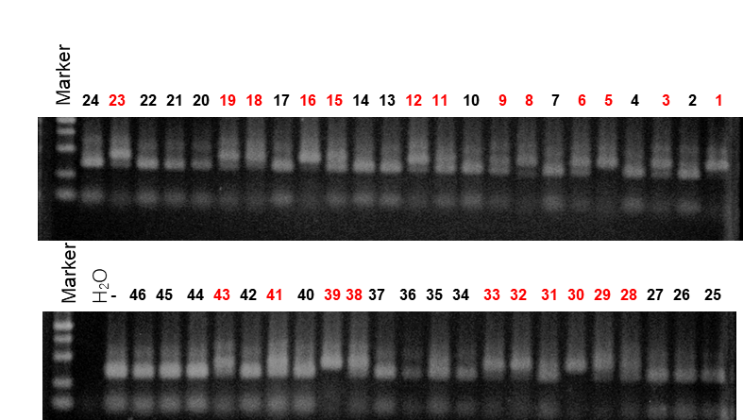
**STU-tRNA-sgRNA3 STU-tRNA-sgRNA**
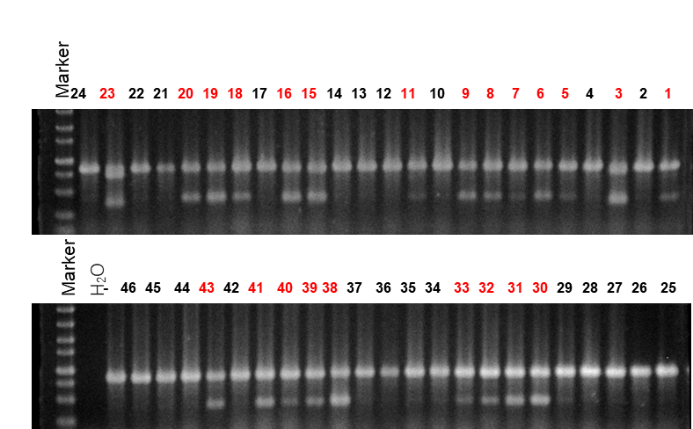
**4**

**STU-Csy4-sgRNA1 STU-Csy4-sgRNA2**


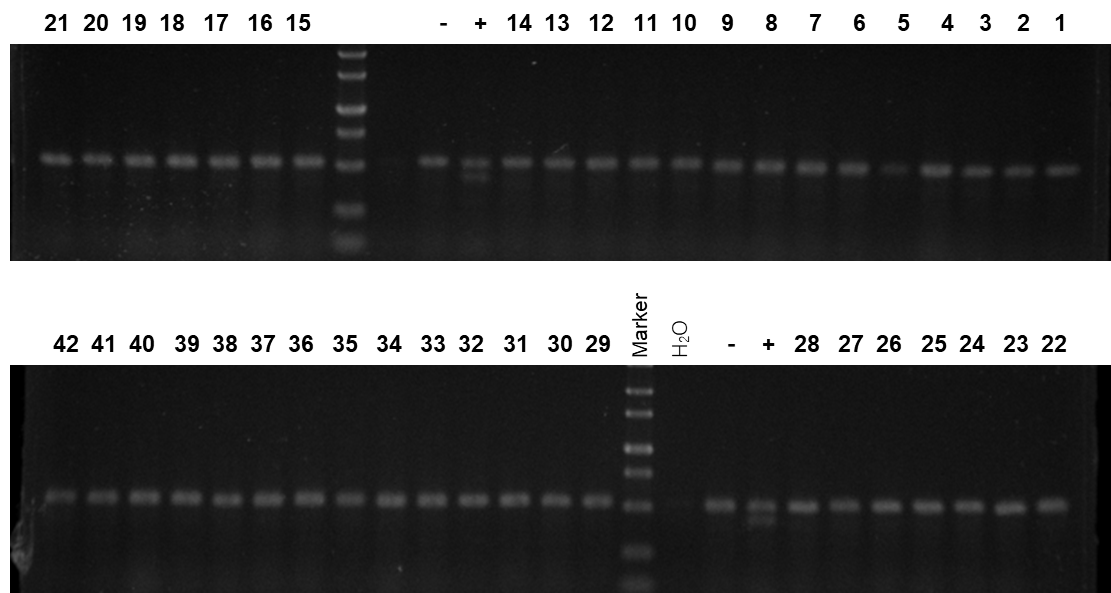

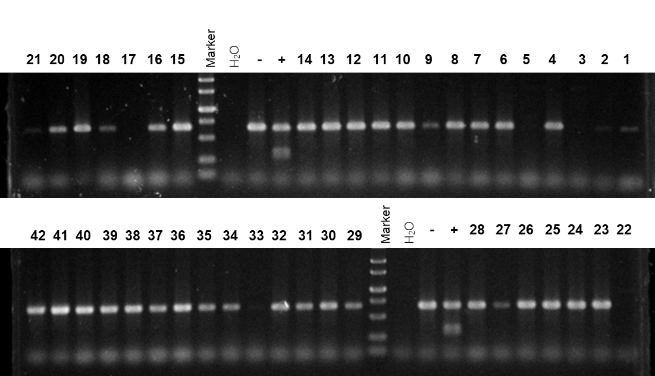


**STU-Csy4-sgRNA3 STU-Csy4-sgRNA4**


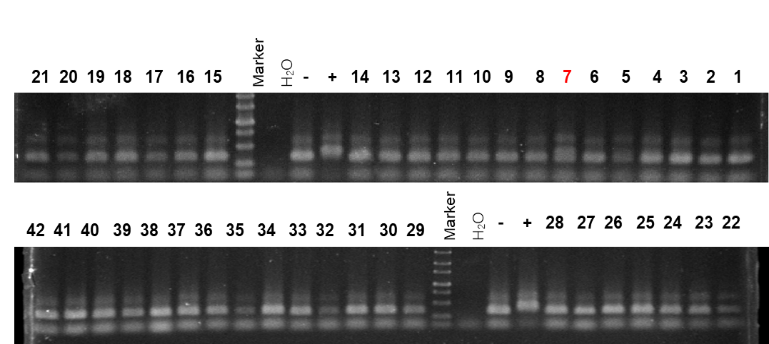

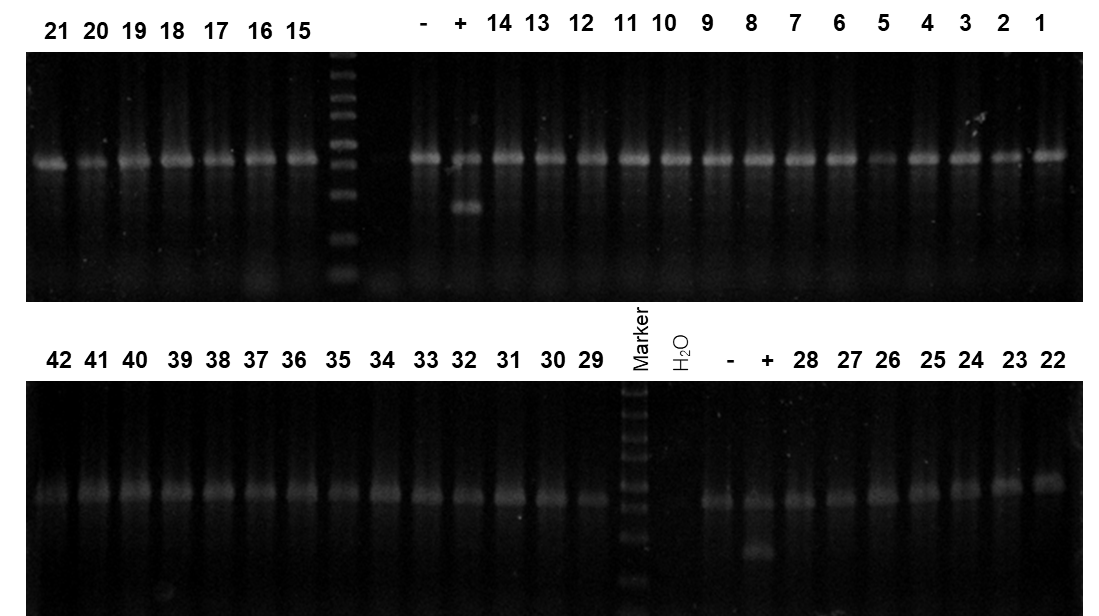


**Supplementary Figure** **4.** Induced mutation analysis using agarose gel electrophoresis of the T7EI or *Pst* I treated PCR products for different target sites. Gene editing was detected in hairy roots. Samples labeled by “red color” were considered mutants. Targeted PCR products of sgRNA1, 2, 3 were treated with T7EI while targeted PCR product of sgRNA4 was treated with enzyme *Pst* I.

**TCTU TCTU-tRNA**


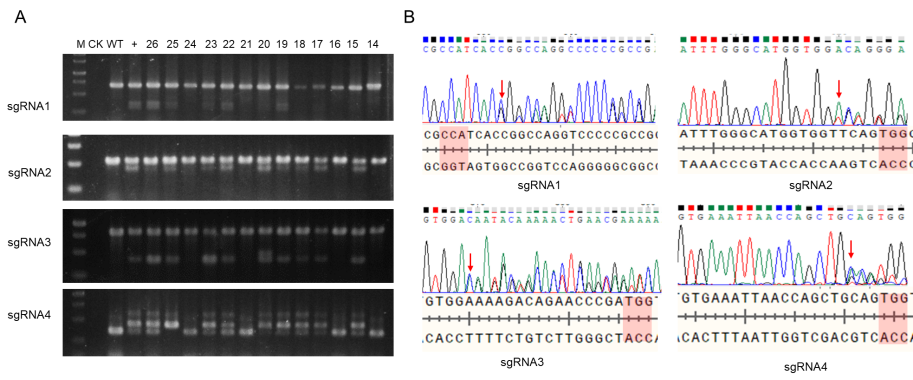

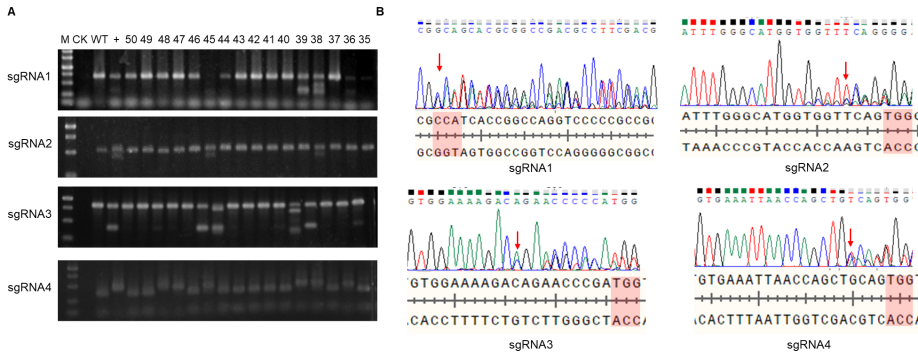


**TCTU-Csy4 STU-tRNA**


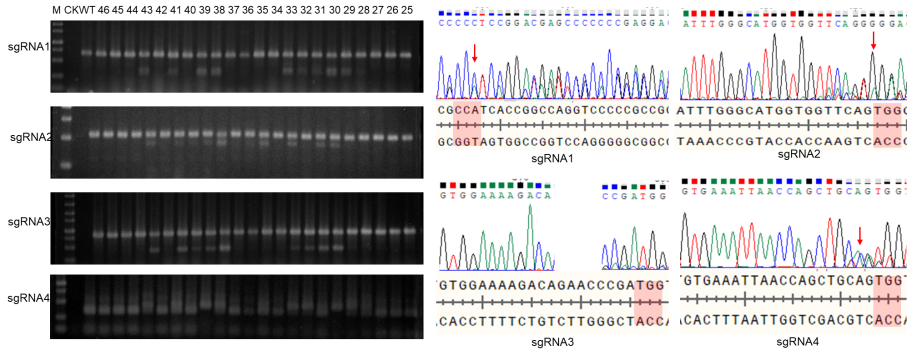
**
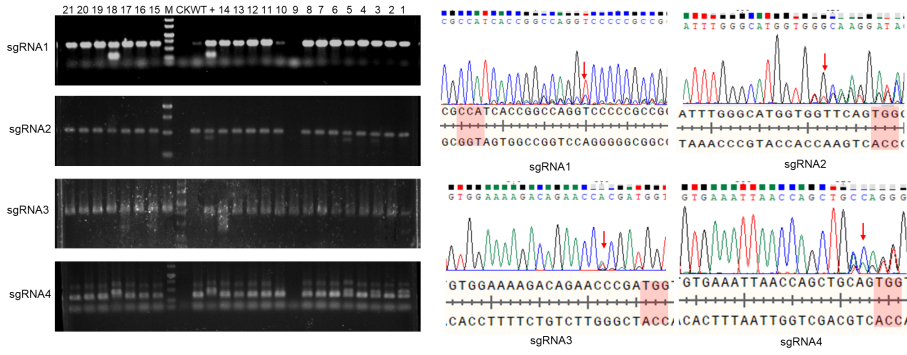
**

**Supplementary Figure** **5.** Sanger sequencing of different gene editing system in soybean hairy root.


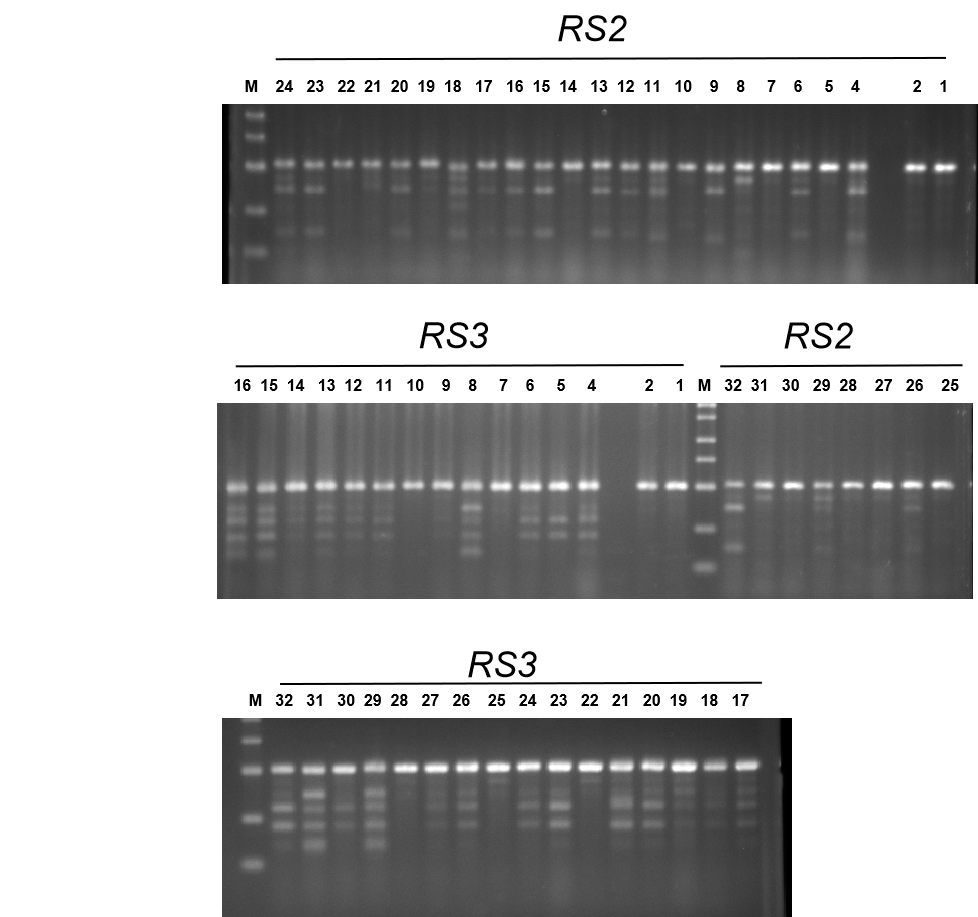


**Supplementary Figure** **6.** Identification of TCTU-tRNA-RS editing efficiency by T7EI in hairy root.


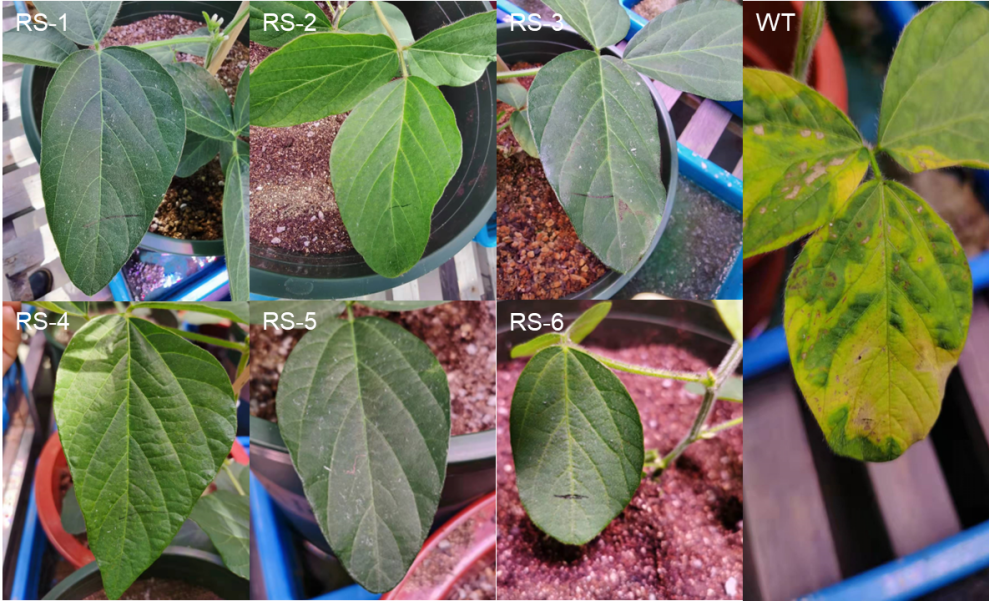


**Supplementary Figure** **7.** Transgenic plants identification by leaf painting with glufosinate.


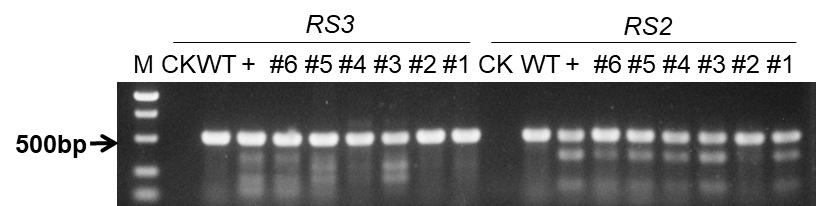


**Supplementary Figure** **8.** Identification of *RS2* or *RS3* mutation in the transgenic plants by T7EI.


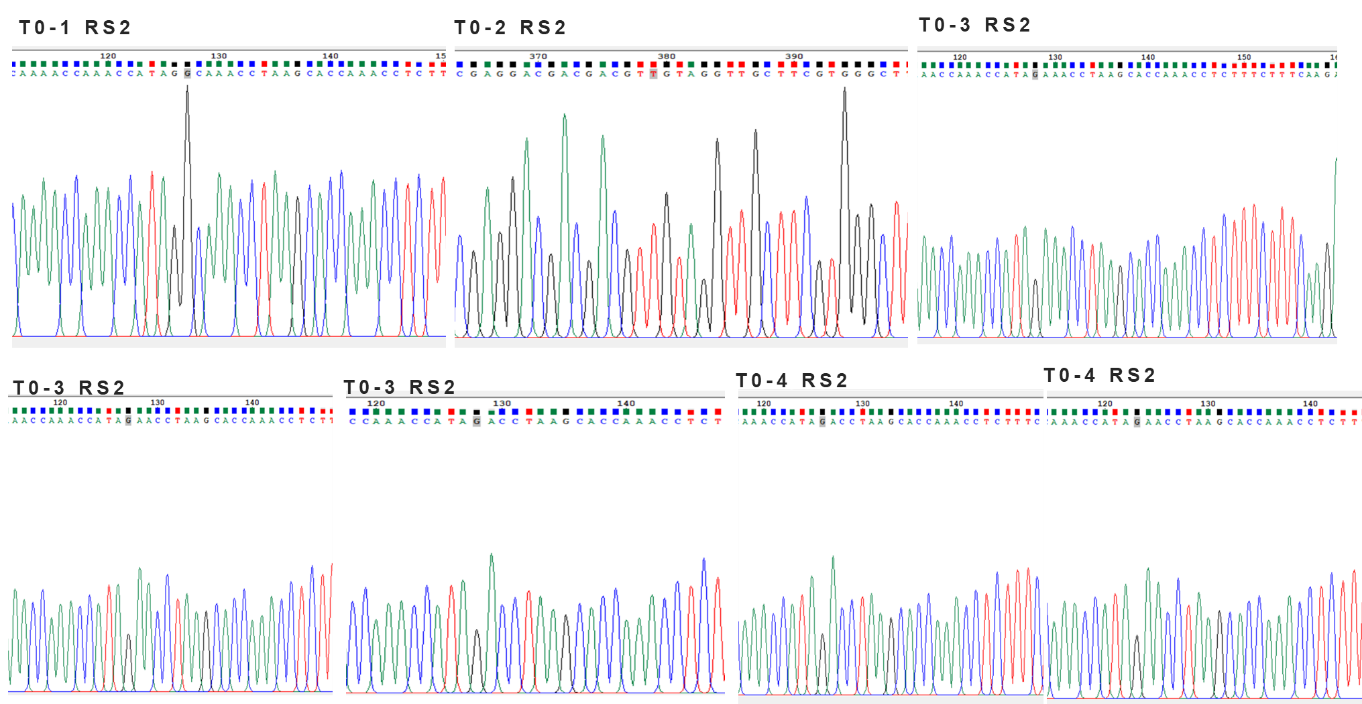


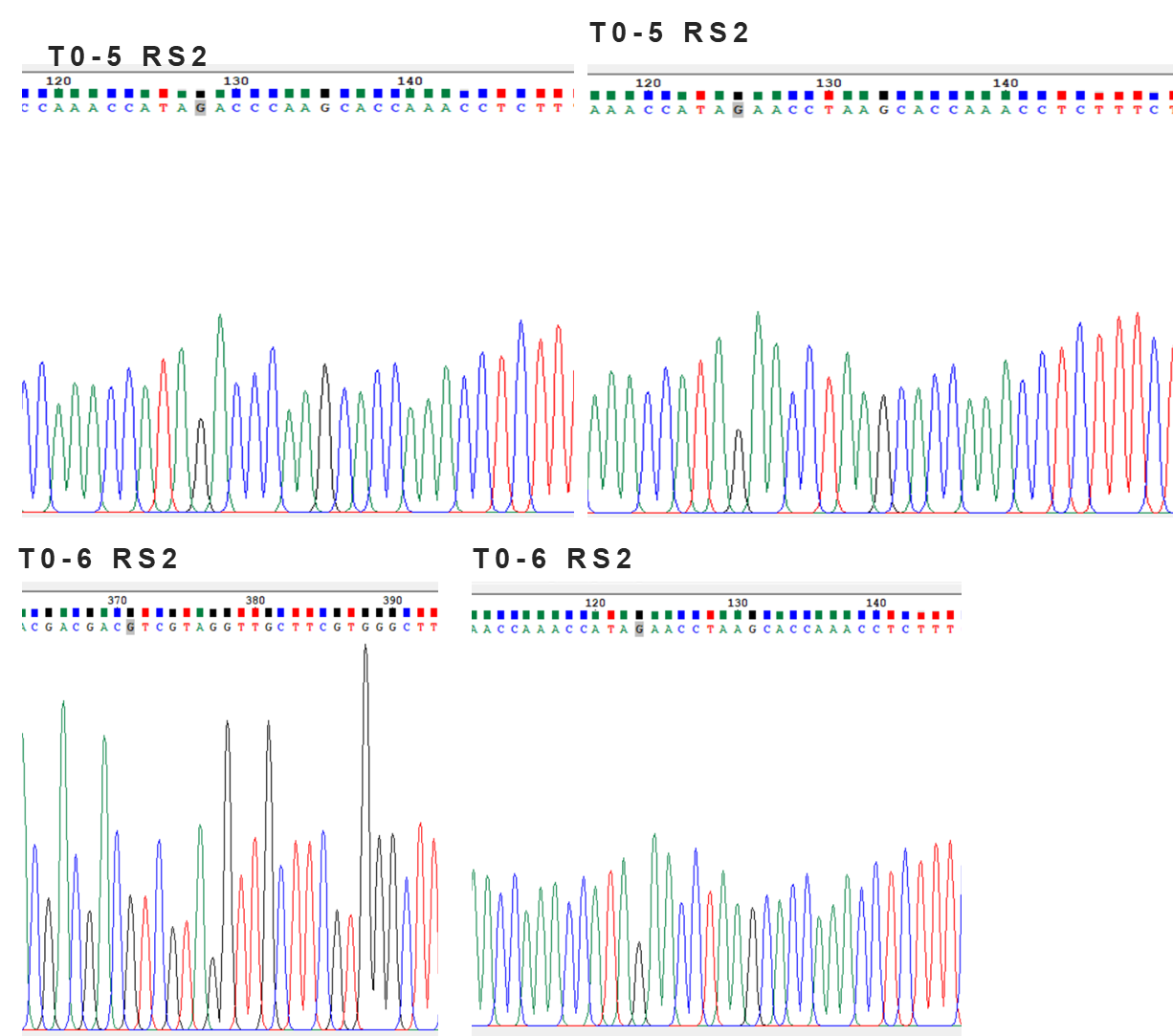


Continued


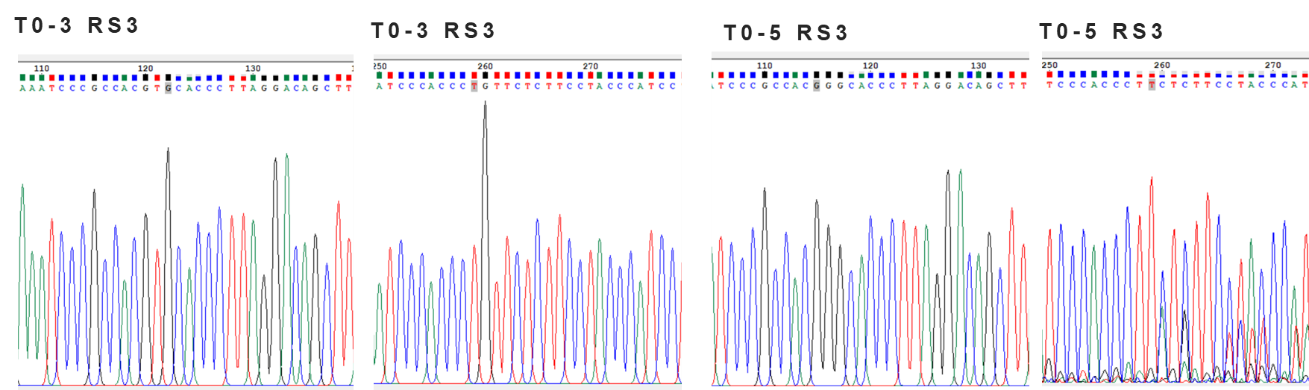


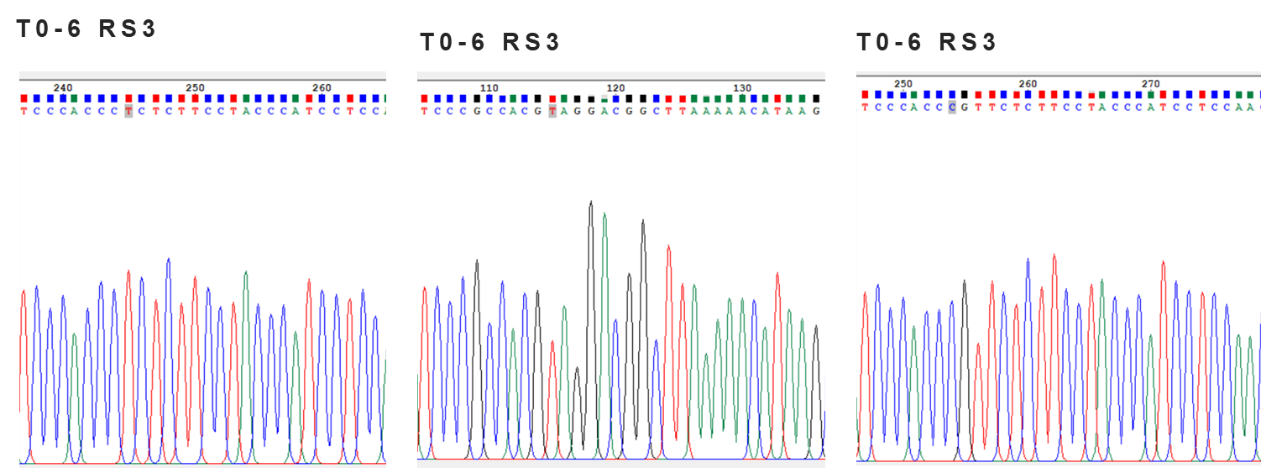


**Supplementary Figure** **9.** Identification of *RS2* or *RS3* mutation in the transgenic plants by Sanger sequencing.

**Supplementary Table 1.** sgRNAs used in this study.

| sgRNA name | Sequence |
| --- | --- |
| sgRNA1 | GCGGGGGACCTGGCCGGTGA |
| sgRNA2 | ATTTGGGCATGGTGGTTCAG |
| sgRNA3 | GTGGAAAAGACAGAACCCGA |
| sgRNA4 | GTGAAATTAACCAGCTGCAG |
| RS3-sgRNA1 | GTAGGAAGAGAACATAAGGG |
| RS3-sgRNA2 | GCTGTCCTAAGGGTGCCACG |
| RS2-sgRNA3 | TTTGGTGCTTAGGTTTGCTA |
| RS2-sgRNA4 | CGACGTCGTAGGTTGCTTCG |

**Supplementary Table 2.** Primers used in this study

| Primer name | Sequence | Purpose |
| --- | --- | --- |
| sgRNA1-F2 | CGGCGGCGCCCTTCACCAAG | Amplifying the sequences of sgRNA1 target gene |
| sgRNA1-R2 | TGAGTCTGACAATGTGTTCC | Amplifying the sequences of sgRNA1 target gene |
| sgRNA2-F | AGAAAAAAAGAGTGGGGGGT | Amplifying the sequences of sgRNA2 target gene |
| sgRNA2-R | CATGTTTGGGAATATCTCAT | Amplifying the sequences of sgRNA2 target gene |
| sgRNA3-F | AGGCTGCATAATAACTCTCG | Amplifying the sequences of sgRNA3 target gene |
| sgRNA3-R | TGCACTCACTGAAGGTATAT | Amplifying the sequences of sgRNA3 target gene |
| sgRNA4-F | GGAGCACTCCACCATCATCTAC | Amplifying the sequences of sgRNA4 target gene |
| sgRNA4-R | GTTCTGACCTCAAACCTTCAAA | Amplifying the sequences of sgRNA4 target gene |
| Cas9-F | ttctggaagccaagggctac | Amplifying the sequences of Cas9 |
| Cas9-R | gatccgtgtctcgtacaggc | Amplifying the sequences of Cas9 |
| Bar-F | gaatcgatgagcccagaacg | Amplifying the sequences of *bar* |
| Bar-R | tcaaatctcggtgacgggca | Amplifying the sequences of *bar* |
| RS2-F | AGAATGCACTAGGCACAATT | Amplifying the sequences of RS2; |
| RS2-R | GTGGTCCACCACACCTTAAA | Amplifying the sequences of RS2 |
| RS3-F | CTGCAACTCAACCCTAAAAG | Amplifying the sequences of RS3; |
| RS3-R | TGCAAGTAGACGACAGTGTC | Amplifying the sequences of RS3 |

**Supplementary Sequence**

DNA sequences of the different gene editing system used in this study. The GmU6, sgRNA, tRNA, GmUBQ, poly T, poly A, Cas9, NOS, P2A, Csy4, spacer and gRNA scaffold are highlighted in orange, dark slate blue, blue, green, deep sky blue, red, purple, gray, pink, Chocolate and black respectively.

TCTU (GmU6-sgRNA1-poly T-GmU6-sgRNA2-poly T-GmU6-sgRNA3-poly T-GmU6-sgRNA4-poly T-GmUBQ-Cas9-NOS)

aaaataaatggtaaaatgtcaaatcaaaactaggctgcagtatgcagagcagagtcatgatgatactacttactacaccgattcttgtgtgcagaaaaatatgttaaaataattgaatctttctctagccaaatttgacaacaatgtacaccgttcatattgagagacgatgcttcttgtttgctttcggtggaagctgcatatactcaacattactccttcagcgagttttccaactgagtcccacattgcccagacctaacacggtattcttgtttataatgaaatgtgccaccacatggattgGCGGGGGACCTGGCCGGTGAgttttagagctagaaatagcaagttaaaataaggctagtccgttatcaacttgaaaaagtggcaccgagtcggtgcttttttttaaaataaatggtaaaatgtcaaatcaaaactaggctgcagtatgcagagcagagtcatgatgatactacttactacaccgattcttgtgtgcagaaaaatatgttaaaataattgaatctttctctagccaaatttgacaacaatgtacaccgttcatattgagagacgatgcttcttgtttgctttcggtggaagctgcatatactcaacattactccttcagcgagttttccaactgagtcccacattgcccagacctaacacggtattcttgtttataatgaaatgtgccaccacatggattgATTTGGGCATGGTGGTTCAGgttttagagctagaaatagcaagttaaaataaggctagtccgttatcaacttgaaaaagtggcaccgagtcggtgcttttttttaaaataaatggtaaaatgtcaaatcaaaactaggctgcagtatgcagagcagagtcatgatgatactacttactacaccgattcttgtgtgcagaaaaatatgttaaaataattgaatctttctctagccaaatttgacaacaatgtacaccgttcatattgagagacgatgcttcttgtttgctttcggtggaagctgcatatactcaacattactccttcagcgagttttccaactgagtcccacattgcccagacctaacacggtattcttgtttataatgaaatgtgccaccacatggattgGTGGAAAAGACAGAACCCGAgttttagagctagaaatagcaagttaaaataaggctagtccgttatcaacttgaaaaagtggcaccgagtcggtgcttttttttaaaataaatggtaaaatgtcaaatcaaaactaggctgcagtatgcagagcagagtcatgatgatactacttactacaccgattcttgtgtgcagaaaaatatgttaaaataattgaatctttctctagccaaatttgacaacaatgtacaccgttcatattgagagacgatgcttcttgtttgctttcggtggaagctgcatatactcaacattactccttcagcgagttttccaactgagtcccacattgcccagacctaacacggtattcttgtttataatgaaatgtgccaccacatggattgGTGAAATTAACCAGCTGCAGgttttagagctagaaatagcaagttaaaataaggctagtccgttatcaacttgaaaaagtggcaccgagtcggtgctttttttttccttaagttgcagcatttaacacatctcctcattagagaaaaaaattcttccctaaacgatagtaaaaacatctaataagaaataagaaagaaaaattaggaaaaagaaaagttcattaaaaaaatcttttggattatttttaaaaaaatatctaaatattttttaaatgaataattttatataaactgtaactaaaagtatacaagtaatgtatgttaacaaaatacttgaaaaatctactgaaaatatatcttacaaagtgaaattaaataagaaagaatttagtggaataattatgattttatttaaaaaataattattaaagatttttttgctccataataagaaaacttttcaattattcttttctggtccataataaaaaaaatctagcatgacagcttttccatagatttttaataatgtaaaagcagccgacttcaggcaatggatagtggggcccgtatcaacttcggacgctccacttgcaacggggtgggcccaatataacaacgacgtcgtaacagataaagcggagcttgaaggtgcatgtgactccgtcaagattacgaaaccgccaactaccacgcaaattgcaattctcaatttcctagaaggactctccgaaaatgcatccaataccaaatattacccgtgtcataggcaccaagtgacaccatacatgaacacgcgtcacaatatgactggagaagggttccacaccttatgctataaaacgccccacacccctcctccttccttcgcagttcaattccaatatattccattctctctgtgtatttccctacctctcccttcaaggttagtcgatttcttctgtttttcttcttcgttctttccatgaattgtgtatgttctttgatcaatacgatgttgatttgattgtgttttgtttggtttcatcgatcttcaattttcataatcagattcagcttttattatctttacaacaacgtccttaatttgatgattctttaatcgtagatttgctctaattagagctttttcatgtcagatccctttacaacaagccttaattgttgattcattaatcgtagattagggcttttttcattgattacttcagatccgttaaacgtaaccatagatcagggctttttcatgaattacttcagatccgttaaacaacagccttattttttatacttctgtggtttttcaagaaattgttcagatccgttgacaaaaagccttattcgttgattctatatcgtttttcgagagatattgctcagatctgttagcaactgccttgtttgttgattctattgccgtggattagggttttttttcacgagattgcttcagatccgtacttaagattacgtaatggattttgattctgatttatctgtgattgttgactcgacagaATGgacaagaagtacagcatcggcctggacatcggcaccaactctgtgggctgggccgtgatcaccgacgagtacaaggtgcccagcaagaaattcaaggtgctgggcaacaccgaccggcacagcatcaagaagaacctgatcggagccctgctgttcgacagcggcgaaacagccgaggccacccggctgaagagaaccgccagaagaagatacaccagacggaagaaccggatctgctatctgcaagagatcttcagcaacgagatggccaaggtggacgacagcttcttccacagactggaagagtccttcctggtggaagaggataagaagcacgagcggcaccccatcttcggcaacatcgtggacgaggtggcctaccacgagaagtaccccaccatctaccacctgagaaagaaactggtggacagcaccgacaaggccgacctgcggctgatctatctggccctggcccacatgatcaagttccggggccacttcctgatcgagggcgacctgaaccccgacaacagcgacgtggacaagctgttcatccagctggtgcagacctacaaccagctgttcgaggaaaaccccatcaacgccagcggcgtggacgccaaggccatcctgtctgccagactgagcaagagcagacggctggaaaatctgatcgcccagctgcccggcgagaagaagaatggcctgttcggaaacctgattgccctgagcctgggcctgacccccaacttcaagagcaacttcgacctggccgaggatgccaaactgcagctgagcaaggacacctacgacgacgacctggacaacctgctggcccagatcggcgaccagtacgccgacctgtttctggccgccaagaacctgtccgacgccatcctgctgagcgacatcctgagagtgaacaccgagatcaccaaggcccccctgagcgcctctatgatcaagagatacgacgagcaccaccaggacctgaccctgctgaaagctctcgtgcggcagcagctgcctgagaagtacaaagagattttcttcgaccagagcaagaacggctacgccggctacattgacggcggagccagccaggaagagttctacaagttcatcaagcccatcctggaaaagatggacggcaccgaggaactgctcgtgaagctgaacagagaggacctgctgcggaagcagcggaccttcgacaacggcagcatcccccaccagatccacctgggagagctgcacgccattctgcggcggcaggaagatttttacccattcctgaaggacaaccgggaaaagatcgagaagatcctgaccttccgcatcccctactacgtgggccctctggccaggggaaacagcagattcgcctggatgaccagaaagagcgaggaaaccatcaccccctggaacttcgaggaagtggtggacaagggcgcttccgcccagagcttcatcgagcggatgaccaacttcgataagaacctgcccaacgagaaggtgctgcccaagcacagcctgctgtacgagtacttcaccgtgtataacgagctgaccaaagtgaaatacgtgaccgagggaatgagaaagcccgccttcctgagcggcgagcagaaaaaggccatcgtggacctgctgttcaagaccaaccggaaagtgaccgtgaagcagctgaaagaggactacttcaagaaaatcgagtgcttcgactccgtggaaatctccggcgtggaagatcggttcaacgcctccctgggcacataccacgatctgctgaaaattatcaaggacaaggacttcctggacaatgaggaaaacgaggacattctggaagatatcgtgctgaccctgacactgtttgaggacagagagatgatcgaggaacggctgaaaacctatgcccacctgttcgacgacaaagtgatgaagcagctgaagcggcggagatacaccggctggggcaggctgagccggaagctgatcaacggcatccgggacaagcagtccggcaagacaatcctggatttcctgaagtccgacggcttcgccaacagaaacttcatgcagctgatccacgacgacagcctgacctttaaagaggacatccagaaagcccaggtgtccggccagggcgatagcctgcacgagcacattgccaatctggccggcagccccgccattaagaagggcatcctgcagacagtgaaggtggtggacgagctcgtgaaagtgatgggccggcacaagcccgagaacatcgtgatcgaaatggccagagagaaccagaccacccagaagggacagaagaacagccgcgagagaatgaagcggatcgaagagggcatcaaagagctgggcagccagatcctgaaagaacaccccgtggaaaacacccagctgcagaacgagaagctgtacctgtactacctgcagaatgggcgggatatgtacgtggaccaggaactggacatcaaccggctgtccgactacgatgtggaccatatcgtgcctcagagctttctgaaggacgactccatcgacaacaaggtgctgaccagaagcgacaagaaccggggcaagagcgacaacgtgccctccgaagaggtcgtgaagaagatgaagaactactggcggcagctgctgaacgccaagctgattacccagagaaagttcgacaatctgaccaaggccgagagaggcggcctgagcgaactggataaggccggcttcatcaagagacagctggtggaaacccggcagatcacaaagcacgtggcacagatcctggactcccggatgaacactaagtacgacgagaatgacaagctgatccgggaagtgaaagtgatcaccctgaagtccaagctggtgtccgatttccggaaggatttccagttttacaaagtgcgcgagatcaacaactaccaccacgcccacgacgcctacctgaacgccgtcgtgggaaccgccctgatcaaaaagtaccctaagctggaaagcgagttcgtgtacggcgactacaaggtgtacgacgtgcggaagatgatcgccaagagcgagcaggaaatcggcaaggctaccgccaagtacttcttctacagcaacatcatgaactttttcaagaccgagattaccctggccaacggcgagatccggaagcggcctctgatcgagacaaacggcgaaaccggggagatcgtgtgggataagggccgggattttgccaccgtgcggaaagtgctgagcatgccccaagtgaatatcgtgaaaaagaccgaggtgcagacaggcggcttcagcaaagagtctatcctgcccaagaggaacagcgataagctgatcgccagaaagaaggactgggaccctaagaagtacggcggcttcgacagccccaccgtggcctattctgtgctggtggtggccaaagtggaaaagggcaagtccaagaaactgaagagtgtgaaagagctgctggggatcaccatcatggaaagaagcagcttcgagaagaatcccatcgactttctggaagccaagggctacaaagaagtgaaaaaggacctgatcatcaagctgcctaagtactccctgttcgagctggaaaacggccggaagagaatgctggcctctgccggcgaactgcagaagggaaacgaactggccctgccctccaaatatgtgaacttcctgtacctggccagccactatgagaagctgaagggctcccccgaggataatgagcagaaacagctgtttgtggaacagcacaagcactacctggacgagatcatcgagcagatcagcgagttctccaagagagtgatcctggccgacgctaatctggacaaagtgctgtccgcctacaacaagcaccgggataagcccatcagagagcaggccgagaatatcatccacctgtttaccctgaccaatctgggagcccctgccgccttcaagtactttgacaccaccatcgaccggaagaggtacaccagcaccaaagaggtgctggacgccaccctgatccaccagagcatcaccggcctgtacgagacacggatcgacctgtctcagctgggaggcgacgatcgttcaaacatttggcaataaagtttcttaagattgaatcctgttgccggtcttgcgatgattatcatataatttctgttgaattacgttaagcatgtaataattaacatgtaatgcatgacgttatttatgagatgggtttttatgattagagtcccgcaattatacatttaatacgcgatagaaaacaaaatatagcgcgcaaactaggataaattatcgcgcgcggtgtcatctatgttactagatc

TCTU-tRNA(GmU6-tRNA-sgRNA1-tRNA-sgRNA2-tRNA-sgRNA3-tRNA-sgRNA4- Poly T-GmUBQ-Cas9-NOS)

AaaataaatggtaaaatgtcaaatcaaaactaggctgcagtatgcagagcagagtcatgatgatactacttactacaccgattcttgtgtgcagaaaaatatgttaaaataattgaatctttctctagccaaatttgacaacaatgtacaccgttcatattgagagacgatgcttcttgtttgctttcggtggaagctgcatatactcaacattactccttcagcgagttttccaactgagtcccacattgcccagacctaacacggtattcttgtttataatgaaatgtgccaccacatggattggtagtaacaAacaaagcaccagtggtctagtggtagaatagtaccctgccacggtacagacccgggttcgattcccggctggtgcaGCGGGGGACCTGGCCGGTGAGttttagagctagaaatagcaagttaaaataaggctagtccgttatcaacttgaaaaagtggcaccgagtcggtgcAacaaagcaccagtggtctagtggtagaatagtaccctgccacggtacagacccgggttcgattcccggctggtgcaATTTGGGCATGGTGGTTCAGGttttagagctagaaatagcaagttaaaataaggctagtccgttatcaacttgaaaaagtggcaccgagtcggtgcAacaaagcaccagtggtctagtggtagaatagtaccctgccacggtacagacccgggttcgattcccggctggtgcaGTGGAAAAGACAGAACCCGAGttttagagctagaaatagcaagttaaaataaggctagtccgttatcaacttgaaaaagtggcaccgagtcggtgcAacaaagcaccagtggtctagtggtagaatagtaccctgccacggtacagacccgggttcgattcccggctggtgcaGTGAAATTAACCAGCTGCAGGttttagagctagaaatagcaagttaaaataaggctagtccgttatcaacttgaaaaagtggcaccgagtcggtgcAacaaagcaccagtggtctagtggtagaatagtaccctgccacggtacagacccgggttcgattcccggctggtgcattttttttcccgggtccttaagttgcagcatttaacacatctcctcattagagaaaaaaattcttccctaaacgatagtaaaaacatctaataagaaataagaaagaaaaattaggaaaaagaaaagttcattaaaaaaatcttttggattatttttaaaaaaatatctaaatattttttaaatgaataattttatataaactgtaactaaaagtatacaagtaatgtatgttaacaaaatacttgaaaaatctactgaaaatatatcttacaaagtgaaattaaataagaaagaatttagtggaataattatgattttatttaaaaaataattattaaagatttttttgctccataataagaaaacttttcaattattcttttctggtccataataaaaaaaatctagcatgacagcttttccatagatttttaataatgtaaaagcagccgacttcaggcaatggatagtggggcccgtatcaacttcggacgctccacttgcaacggggtgggcccaatataacaacgacgtcgtaacagataaagcggagcttgaaggtgcatgtgactccgtcaagattacgaaaccgccaactaccacgcaaattgcaattctcaatttcctagaaggactctccgaaaatgcatccaataccaaatattacccgtgtcataggcaccaagtgacaccatacatgaacacgcgtcacaatatgactggagaagggttccacaccttatgctataaaacgccccacacccctcctccttccttcgcagttcaattccaatatattccattctctctgtgtatttccctacctctcccttcaaggttagtcgatttcttctgtttttcttcttcgttctttccatgaattgtgtatgttctttgatcaatacgatgttgatttgattgtgttttgtttggtttcatcgatcttcaattttcataatcagattcagcttttattatctttacaacaacgtccttaatttgatgattctttaatcgtagatttgctctaattagagctttttcatgtcagatccctttacaacaagccttaattgttgattcattaatcgtagattagggcttttttcattgattacttcagatccgttaaacgtaaccatagatcagggctttttcatgaattacttcagatccgttaaacaacagccttattttttatacttctgtggtttttcaagaaattgttcagatccgttgacaaaaagccttattcgttgattctatatcgtttttcgagagatattgctcagatctgttagcaactgccttgtttgttgattctattgccgtggattagggttttttttcacgagattgcttcagatccgtacttaagattacgtaatggattttgattctgatttatctgtgattgttgactcgacagaATGgacaagaagtacagcatcggcctggacatcggcaccaactctgtgggctgggccgtgatcaccgacgagtacaaggtgcccagcaagaaattcaaggtgctgggcaacaccgaccggcacagcatcaagaagaacctgatcggagccctgctgttcgacagcggcgaaacagccgaggccacccggctgaagagaaccgccagaagaagatacaccagacggaagaaccggatctgctatctgcaagagatcttcagcaacgagatggccaaggtggacgacagcttcttccacagactggaagagtccttcctggtggaagaggataagaagcacgagcggcaccccatcttcggcaacatcgtggacgaggtggcctaccacgagaagtaccccaccatctaccacctgagaaagaaactggtggacagcaccgacaaggccgacctgcggctgatctatctggccctggcccacatgatcaagttccggggccacttcctgatcgagggcgacctgaaccccgacaacagcgacgtggacaagctgttcatccagctggtgcagacctacaaccagctgttcgaggaaaaccccatcaacgccagcggcgtggacgccaaggccatcctgtctgccagactgagcaagagcagacggctggaaaatctgatcgcccagctgcccggcgagaagaagaatggcctgttcggaaacctgattgccctgagcctgggcctgacccccaacttcaagagcaacttcgacctggccgaggatgccaaactgcagctgagcaaggacacctacgacgacgacctggacaacctgctggcccagatcggcgaccagtacgccgacctgtttctggccgccaagaacctgtccgacgccatcctgctgagcgacatcctgagagtgaacaccgagatcaccaaggcccccctgagcgcctctatgatcaagagatacgacgagcaccaccaggacctgaccctgctgaaagctctcgtgcggcagcagctgcctgagaagtacaaagagattttcttcgaccagagcaagaacggctacgccggctacattgacggcggagccagccaggaagagttctacaagttcatcaagcccatcctggaaaagatggacggcaccgaggaactgctcgtgaagctgaacagagaggacctgctgcggaagcagcggaccttcgacaacggcagcatcccccaccagatccacctgggagagctgcacgccattctgcggcggcaggaagatttttacccattcctgaaggacaaccgggaaaagatcgagaagatcctgaccttccgcatcccctactacgtgggccctctggccaggggaaacagcagattcgcctggatgaccagaaagagcgaggaaaccatcaccccctggaacttcgaggaagtggtggacaagggcgcttccgcccagagcttcatcgagcggatgaccaacttcgataagaacctgcccaacgagaaggtgctgcccaagcacagcctgctgtacgagtacttcaccgtgtataacgagctgaccaaagtgaaatacgtgaccgagggaatgagaaagcccgccttcctgagcggcgagcagaaaaaggccatcgtggacctgctgttcaagaccaaccggaaagtgaccgtgaagcagctgaaagaggactacttcaagaaaatcgagtgcttcgactccgtggaaatctccggcgtggaagatcggttcaacgcctccctgggcacataccacgatctgctgaaaattatcaaggacaaggacttcctggacaatgaggaaaacgaggacattctggaagatatcgtgctgaccctgacactgtttgaggacagagagatgatcgaggaacggctgaaaacctatgcccacctgttcgacgacaaagtgatgaagcagctgaagcggcggagatacaccggctggggcaggctgagccggaagctgatcaacggcatccgggacaagcagtccggcaagacaatcctggatttcctgaagtccgacggcttcgccaacagaaacttcatgcagctgatccacgacgacagcctgacctttaaagaggacatccagaaagcccaggtgtccggccagggcgatagcctgcacgagcacattgccaatctggccggcagccccgccattaagaagggcatcctgcagacagtgaaggtggtggacgagctcgtgaaagtgatgggccggcacaagcccgagaacatcgtgatcgaaatggccagagagaaccagaccacccagaagggacagaagaacagccgcgagagaatgaagcggatcgaagagggcatcaaagagctgggcagccagatcctgaaagaacaccccgtggaaaacacccagctgcagaacgagaagctgtacctgtactacctgcagaatgggcgggatatgtacgtggaccaggaactggacatcaaccggctgtccgactacgatgtggaccatatcgtgcctcagagctttctgaaggacgactccatcgacaacaaggtgctgaccagaagcgacaagaaccggggcaagagcgacaacgtgccctccgaagaggtcgtgaagaagatgaagaactactggcggcagctgctgaacgccaagctgattacccagagaaagttcgacaatctgaccaaggccgagagaggcggcctgagcgaactggataaggccggcttcatcaagagacagctggtggaaacccggcagatcacaaagcacgtggcacagatcctggactcccggatgaacactaagtacgacgagaatgacaagctgatccgggaagtgaaagtgatcaccctgaagtccaagctggtgtccgatttccggaaggatttccagttttacaaagtgcgcgagatcaacaactaccaccacgcccacgacgcctacctgaacgccgtcgtgggaaccgccctgatcaaaaagtaccctaagctggaaagcgagttcgtgtacggcgactacaaggtgtacgacgtgcggaagatgatcgccaagagcgagcaggaaatcggcaaggctaccgccaagtacttcttctacagcaacatcatgaactttttcaagaccgagattaccctggccaacggcgagatccggaagcggcctctgatcgagacaaacggcgaaaccggggagatcgtgtgggataagggccgggattttgccaccgtgcggaaagtgctgagcatgccccaagtgaatatcgtgaaaaagaccgaggtgcagacaggcggcttcagcaaagagtctatcctgcccaagaggaacagcgataagctgatcgccagaaagaaggactgggaccctaagaagtacggcggcttcgacagccccaccgtggcctattctgtgctggtggtggccaaagtggaaaagggcaagtccaagaaactgaagagtgtgaaagagctgctggggatcaccatcatggaaagaagcagcttcgagaagaatcccatcgactttctggaagccaagggctacaaagaagtgaaaaaggacctgatcatcaagctgcctaagtactccctgttcgagctggaaaacggccggaagagaatgctggcctctgccggcgaactgcagaagggaaacgaactggccctgccctccaaatatgtgaacttcctgtacctggccagccactatgagaagctgaagggctcccccgaggataatgagcagaaacagctgtttgtggaacagcacaagcactacctggacgagatcatcgagcagatcagcgagttctccaagagagtgatcctggccgacgctaatctggacaaagtgctgtccgcctacaacaagcaccgggataagcccatcagagagcaggccgagaatatcatccacctgtttaccctgaccaatctgggagcccctgccgccttcaagtactttgacaccaccatcgaccggaagaggtacaccagcaccaaagaggtgctggacgccaccctgatccaccagagcatcaccggcctgtacgagacacggatcgacctgtctcagctgggaggcgacgatcgttcaaacatttggcaataaagtttcttaagattgaatcctgttgccggtcttgcgatgattatcatataatttctgttgaattacgttaagcatgtaataattaacatgtaatgcatgacgttatttatgagatgggtttttatgattagagtcccgcaattatacatttaatacgcgatagaaaacaaaatatagcgcgcaaactaggataaattatcgcgcgcggtgtcatctatgttactagatc

TCTU-Csy4(GmU6-Csy4 cleavage site-sgRNA1-Csy4 cleavage site-sgRNA2-Csy4 cleavage site-sgRNA3-Csy4 cleavage site-sgRNA4-Poly T-GmUBQ-Csy4-P2A-Cas9-NOS)

AaaataaatggtaaaatgtcaaatcaaaactaggctgcagtatgcagagcagagtcatgatgatactacttactacaccgattcttgtgtgcagaaaaatatgttaaaataattgaatctttctctagccaaatttgacaacaatgtacaccgttcatattgagagacgatgcttcttgtttgctttcggtggaagctgcatatactcaacattactccttcagcgagttttccaactgagtcccacattgcccagacctaacacggtattcttgtttataatgaaatgtgccaccacatggattgGtagtaacaGTTCACTGCCGTATAGGCAGGCGGGGGACCTGGCCGGTGAgttttagagctagaaatagcaagttaaaataaggctagtccgttatcaacttgaaaaagtggcaccgagtcggtgcGTTCACTGCCGTATAGGCAGATTTGGGCATGGTGGTTCAGgttttagagctagaaatagcaagttaaaataaggctagtccgttatcaacttgaaaaagtggcaccgagtcggtgcGTTCACTGCCGTATAGGCAGGTGGAAAAGACAGAACCCGAgttttagagctagaaatagcaagttaaaataaggctagtccgttatcaacttgaaaaagtggcaccgagtcggtgcGTTCACTGCCGTATAGGCAGGTGAAATTAACCAGCTGCAGgttttagagctagaaatagcaagttaaaataaggctagtccgttatcaacttgaaaaagtggcaccgagtcggtgcGTTCACTGCCGTATAGGCAGtttttttttccttaagttgcagcatttaacacatctcctcattagagaaaaaaattcttccctaaacgatagtaaaaacatctaataagaaataagaaagaaaaattaggaaaaagaaaagttcattaaaaaaatcttttggattatttttaaaaaaatatctaaatattttttaaatgaataattttatataaactgtaactaaaagtatacaagtaatgtatgttaacaaaatacttgaaaaatctactgaaaatatatcttacaaagtgaaattaaataagaaagaatttagtggaataattatgattttatttaaaaaataattattaaagatttttttgctccataataagaaaacttttcaattattcttttctggtccataataaaaaaaatctagcatgacagcttttccatagatttttaataatgtaaaagcagccgacttcaggcaatggatagtggggcccgtatcaacttcggacgctccacttgcaacggggtgggcccaatataacaacgacgtcgtaacagataaagcgGagcttgaaggtgcatgtgactccgtcaagattacgaaaccgccaactaccacgcaaattgcaattctcaatttcctagaaggactctccgaaaatgcatccaataccaaatattacccgtgtcataggcaccaagtgacaccatacatgaacacgcgtcacaatatgactggagaagggttccacaccttatgctataaaacgccccacacccctcctccttccttcgcagttcaattccaatatattccattctctctgtgtatttccctacctctcccttcaaggttagtcgatttcttctgtttttcttcttcgttctttccatgaattgtgtatgttctttgatcaatacgatgttgatttgattgtgttttgtttggtttcatcgatcttcaattttcataatcagattcagcttttattatctttacaacaacgtccttaatttgatgattctttaatcgtagatttgctctaattagagctttttcatgtcagatccctttacaacaagccttaattgttgattcattaatcgtagattagggcttttttcattgattacttcagatccgttaaacgtaaccatagatcagggctttttcatgaattacttcagatccgttaaacaacagccttattttttatacttctgtggtttttcaagaaattgttcagatccgttgacaaaaagccttattcgttgattctatatcgtttttcgagagatattgctcagatctgttagcaactgccttgtttgttgattctattgccgtggattagggttttttttcacgagattgcttcagatccgtacttaagattacgtaatggattttgattctgatttatctgtgattgttgactcgacagaatggatcattatcttgatattagacttagacctgatccagaatttccaccagctcaacttatgtctgttctttttggaaaacttcatcaagctcttgttgctcaaggaggagatagaattggagtttcttttcctgatcttgatgaatcaagatcaagacttggagaaagacttagaattcatgcttctgctgatgatcttagagctttgcttgctagaccttggcttgaaggacttagagatcatcttcaatttggagaaccagctgttgttccacatccaactccttatagacaagtttcaagagttcaagctaaatctaatccagaaagacttagaagaagacttatgagaagacatgatctttctgaagaagaagctagaaaaagaattcctgatactgttgctagagctttggatttgccttttgttacacttagatcacaatctactggacaacattttagactttttattagacatggaccacttcaagttactgctgaagaaggaggatttacttgttatggactttctaagggaggttttgttccttggtttGGATCTGGTGCTACTAACTTCTCACTTTTGAAGCAAGCAGGAGATGTTGAGGAAAATCCAGGTCCTATGgacaagaagtacagcatcggcctggacatcggcaccaactctgtgggctgggccgtgatcaccgacgagtacaaggtgcccagcaagaaattcaaggtgctgggcaacaccgaccggcacagcatcaagaagaacctgatcggagccctgctgttcgacagcggcgaaacagccgaggccacccggctgaagagaaccgccagaagaagatacaccagacggaagaaccggatctgctatctgcaagagatcttcagcaacgagatggccaaggtggacgacagcttcttccacagactggaagagtccttcctggtggaagaggataagaagcacgagcggcaccccatcttcggcaacatcgtggacgaggtggcctaccacgagaagtaccccaccatctaccacctgagaaagaaactggtggacagcaccgacaaggccgacctgcggctgatctatctggccctggcccacatgatcaagttccggggccacttcctgatcgagggcgacctgaaccccgacaacagcgacgtggacaagctgttcatccagctggtgcagacctacaaccagctgttcgaggaaaaccccatcaacgccagcggcgtggacgccaaggccatcctgtctgccagactgagcaagagcagacggctggaaaatctgatcgcccagctgcccggcgagaagaagaatggcctgttcggaaacctgattgccctgagcctgggcctgacccccaacttcaagagcaacttcgacctggccgaggatgccaaactgcagctgagcaaggacacctacgacgacgacctggacaacctgctggcccagatcggcgaccagtacgccgacctgtttctggccgccaagaacctgtccgacgccatcctgctgagcgacatcctgagagtgaacaccgagatcaccaaggcccccctgagcgcctctatgatcaagagatacgacgagcaccaccaggacctgaccctgctgaaagctctcgtgcggcagcagctgcctgagaagtacaaagagattttcttcgaccagagcaagaacggctacgccggctacattgacggcggagccagccaggaagagttctacaagttcatcaagcccatcctggaaaagatggacggcaccgaggaactgctcgtgaagctgaacagagaggacctgctgcggaagcagcggaccttcgacaacggcagcatcccccaccagatccacctgggagagctgcacgccattctgcggcggcaggaagatttttacccattcctgaaggacaaccgggaaaagatcgagaagatcctgaccttccgcatcccctactacgtgggccctctggccaggggaaacagcagattcgcctggatgaccagaaagagcgaggaaaccatcaccccctggaacttcgaggaagtggtggacaagggcgcttccgcccagagcttcatcgagcggatgaccaacttcgataagaacctgcccaacgagaaggtgctgcccaagcacagcctgctgtacgagtacttcaccgtgtataacgagctgaccaaagtgaaatacgtgaccgagggaatgagaaagcccgccttcctgagcggcgagcagaaaaaggccatcgtggacctgctgttcaagaccaaccggaaagtgaccgtgaagcagctgaaagaggactacttcaagaaaatcgagtgcttcgactccgtggaaatctccggcgtggaagatcggttcaacgcctccctgggcacataccacgatctgctgaaaattatcaaggacaaggacttcctggacaatgaggaaaacgaggacattctggaagatatcgtgctgaccctgacactgtttgaggacagagagatgatcgaggaacggctgaaaacctatgcccacctgttcgacgacaaagtgatgaagcagctgaagcggcggagatacaccggctggggcaggctgagccggaagctgatcaacggcatccgggacaagcagtccggcaagacaatcctggatttcctgaagtccgacggcttcgccaacagaaacttcatgcagctgatccacgacgacagcctgacctttaaagaggacatccagaaagcccaggtgtccggccagggcgatagcctgcacgagcacattgccaatctggccggcagccccgccattaagaagggcatcctgcagacagtgaaggtggtggacgagctcgtgaaagtgatgggccggcacaagcccgagaacatcgtgatcgaaatggccagagagaaccagaccacccagaagggacagaagaacagccgcgagagaatgaagcggatcgaagagggcatcaaagagctgggcagccagatcctgaaagaacaccccgtggaaaacacccagctgcagaacgagaagctgtacctgtactacctgcagaatgggcgggatatgtacgtggaccaggaactggacatcaaccggctgtccgactacgatgtggaccatatcgtgcctcagagctttctgaaggacgactccatcgacaacaaggtgctgaccagaagcgacaagaaccggggcaagagcgacaacgtgccctccgaagaggtcgtgaagaagatgaagaactactggcggcagctgctgaacgccaagctgattacccagagaaagttcgacaatctgaccaaggccgagagaggcggcctgagcgaactggataaggccggcttcatcaagagacagctggtggaaacccggcagatcacaaagcacgtggcacagatcctggactcccggatgaacactaagtacgacgagaatgacaagctgatccgggaagtgaaagtgatcaccctgaagtccaagctggtgtccgatttccggaaggatttccagttttacaaagtgcgcgagatcaacaactaccaccacgcccacgacgcctacctgaacgccgtcgtgggaaccgccctgatcaaaaagtaccctaagctggaaagcgagttcgtgtacggcgactacaaggtgtacgacgtgcggaagatgatcgccaagagcgagcaggaaatcggcaaggctaccgccaagtacttcttctacagcaacatcatgaactttttcaagaccgagattaccctggccaacggcgagatccggaagcggcctctgatcgagacaaacggcgaaaccggggagatcgtgtgggataagggccgggattttgccaccgtgcggaaagtgctgagcatgccccaagtgaatatcgtgaaaaagaccgaggtgcagacaggcggcttcagcaaagagtctatcctgcccaagaggaacagcgataagctgatcgccagaaagaaggactgggaccctaagaagtacggcggcttcgacagccccaccgtggcctattctgtgctggtggtggccaaagtggaaaagggcaagtccaagaaactgaagagtgtgaaagagctgctggggatcaccatcatggaaagaagcagcttcgagaagaatcccatcgactttctggaagccaagggctacaaagaagtgaaaaaggacctgatcatcaagctgcctaagtactccctgttcgagctggaaaacggccggaagagaatgctggcctctgccggcgaactgcagaagggaaacgaactggccctgccctccaaatatgtgaacttcctgtacctggccagccactatgagaagctgaagggctcccccgaggataatgagcagaaacagctgtttgtggaacagcacaagcactacctggacgagatcatcgagcagatcagcgagttctccaagagagtgatcctggccgacgctaatctggacaaagtgctgtccgcctacaacaagcaccgggataagcccatcagagagcaggccgagaatatcatccacctgtttaccctgaccaatctgggagcccctgccgccttcaagtactttgacaccaccatcgaccggaagaggtacaccagcaccaaagaggtgctggacgccaccctgatccaccagagcatcaccggcctgtacgagacacggatcgacctgtctcagctgggaggcgacgatcgttcaaacatttggcaataaagtttcttaagattgaatcctgttgccggtcttgcgatgattatcatataatttctgttgaattacgttaagcatgtaataattaacatgtaatgcatgacgttatttatgagatgggtttttatgattagagtcccgcaattatacatttaatacgcgatagaaaacaaaatatagcgcgcaaactaggataaattatcgcgcgcggtgtcatctatgttactagatc

STU-tRNA(GmUBQ-Cas9-Poly A-tRNA-sgRNA1-tRNA-sgRNA2-tRNA-sgRNA3-tRNA-sgRNA4-NOS)

tccttaagttgcagcatttaacacatctcctcattagagaaaaaaattcttccctaaacgatagtaaaaacatctaataagaaataagaaagaaaaattaggaaaaagaaaagttcattaaaaaaatcttttggattatttttaaaaaaatatctaaatattttttaaatgaataattttatataaactgtaactaaaagtatacaagtaatgtatgttaacaaaatacttgaaaaatctactgaaaatatatcttacaaagtgaaattaaataagaaagaatttagtggaataattatgattttatttaaaaaataattattaaagatttttttgctccataataagaaaacttttcaattattcttttctggtccataataaaaaaaatctagcatgacagcttttccatagatttttaataatgtaaaagcagccgacttcaggcaatggatagtggggcccgtatcaacttcggacgctccacttgcaacggggtgggcccaatataacaacgacgtcgtaacagataaagcgGagcttgaaggtgcatgtgactccgtcaagattacgaaaccgccaactaccacgcaaattgcaattctcaatttcctagaaggactctccgaaaatgcatccaataccaaatattacccgtgtcataggcaccaagtgacaccatacatgaacacgcgtcacaatatgactggagaagggttccacaccttatgctataaaacgccccacacccctcctccttccttcgcagttcaattccaatatattccattctctctgtgtatttccctacctctcccttcaaggttagtcgatttcttctgtttttcttcttcgttctttccatgaattgtgtatgttctttgatcaatacgatgttgatttgattgtgttttgtttggtttcatcgatcttcaattttcataatcagattcagcttttattatctttacaacaacgtccttaatttgatgattctttaatcgtagatttgctctaattagagctttttcatgtcagatccctttacaacaagccttaattgttgattcattaatcgtagattagggcttttttcattgattacttcagatccgttaaacgtaaccatagatcagggctttttcatgaattacttcagatccgttaaacaacagccttattttttatacttctgtggtttttcaagaaattgttcagatccgttgacaaaaagccttattcgttgattctatatcgtttttcgagagatattgctcagatctgttagcaactgccttgtttgttgattctattgccgtggattagggttttttttcacgagattgcttcagatccgtacttaagattacgtaatggattttgattctgatttatctgtgattgttgactcgacagaATGgacaagaagtacagcatcggcctggacatcggcaccaactctgtgggctgggccgtgatcaccgacgagtacaaggtgcccagcaagaaattcaaggtgctgggcaacaccgaccggcacagcatcaagaagaacctgatcggagccctgctgttcgacagcggcgaaacagccgaggccacccggctgaagagaaccgccagaagaagatacaccagacggaagaaccggatctgctatctgcaagagatcttcagcaacgagatggccaaggtggacgacagcttcttccacagactggaagagtccttcctggtggaagaggataagaagcacgagcggcaccccatcttcggcaacatcgtggacgaggtggcctaccacgagaagtaccccaccatctaccacctgagaaagaaactggtggacagcaccgacaaggccgacctgcggctgatctatctggccctggcccacatgatcaagttccggggccacttcctgatcgagggcgacctgaaccccgacaacagcgacgtggacaagctgttcatccagctggtgcagacctacaaccagctgttcgaggaaaaccccatcaacgccagcggcgtggacgccaaggccatcctgtctgccagactgagcaagagcagacggctggaaaatctgatcgcccagctgcccggcgagaagaagaatggcctgttcggaaacctgattgccctgagcctgggcctgacccccaacttcaagagcaacttcgacctggccgaggatgccaaactgcagctgagcaaggacacctacgacgacgacctggacaacctgctggcccagatcggcgaccagtacgccgacctgtttctggccgccaagaacctgtccgacgccatcctgctgagcgacatcctgagagtgaacaccgagatcaccaaggcccccctgagcgcctctatgatcaagagatacgacgagcaccaccaggacctgaccctgctgaaagctctcgtgcggcagcagctgcctgagaagtacaaagagattttcttcgaccagagcaagaacggctacgccggctacattgacggcggagccagccaggaagagttctacaagttcatcaagcccatcctggaaaagatggacggcaccgaggaactgctcgtgaagctgaacagagaggacctgctgcggaagcagcggaccttcgacaacggcagcatcccccaccagatccacctgggagagctgcacgccattctgcggcggcaggaagatttttacccattcctgaaggacaaccgggaaaagatcgagaagatcctgaccttccgcatcccctactacgtgggccctctggccaggggaaacagcagattcgcctggatgaccagaaagagcgaggaaaccatcaccccctggaacttcgaggaagtggtggacaagggcgcttccgcccagagcttcatcgagcggatgaccaacttcgataagaacctgcccaacgagaaggtgctgcccaagcacagcctgctgtacgagtacttcaccgtgtataacgagctgaccaaagtgaaatacgtgaccgagggaatgagaaagcccgccttcctgagcggcgagcagaaaaaggccatcgtggacctgctgttcaagaccaaccggaaagtgaccgtgaagcagctgaaagaggactacttcaagaaaatcgagtgcttcgactccgtggaaatctccggcgtggaagatcggttcaacgcctccctgggcacataccacgatctgctgaaaattatcaaggacaaggacttcctggacaatgaggaaaacgaggacattctggaagatatcgtgctgaccctgacactgtttgaggacagagagatgatcgaggaacggctgaaaacctatgcccacctgttcgacgacaaagtgatgaagcagctgaagcggcggagatacaccggctggggcaggctgagccggaagctgatcaacggcatccgggacaagcagtccggcaagacaatcctggatttcctgaagtccgacggcttcgccaacagaaacttcatgcagctgatccacgacgacagcctgacctttaaagaggacatccagaaagcccaggtgtccggccagggcgatagcctgcacgagcacattgccaatctggccggcagccccgccattaagaagggcatcctgcagacagtgaaggtggtggacgagctcgtgaaagtgatgggccggcacaagcccgagaacatcgtgatcgaaatggccagagagaaccagaccacccagaagggacagaagaacagccgcgagagaatgaagcggatcgaagagggcatcaaagagctgggcagccagatcctgaaagaacaccccgtggaaaacacccagctgcagaacgagaagctgtacctgtactacctgcagaatgggcgggatatgtacgtggaccaggaactggacatcaaccggctgtccgactacgatgtggaccatatcgtgcctcagagctttctgaaggacgactccatcgacaacaaggtgctgaccagaagcgacaagaaccggggcaagagcgacaacgtgccctccgaagaggtcgtgaagaagatgaagaactactggcggcagctgctgaacgccaagctgattacccagagaaagttcgacaatctgaccaaggccgagagaggcggcctgagcgaactggataaggccggcttcatcaagagacagctggtggaaacccggcagatcacaaagcacgtggcacagatcctggactcccggatgaacactaagtacgacgagaatgacaagctgatccgggaagtgaaagtgatcaccctgaagtccaagctggtgtccgatttccggaaggatttccagttttacaaagtgcgcgagatcaacaactaccaccacgcccacgacgcctacctgaacgccgtcgtgggaaccgccctgatcaaaaagtaccctaagctggaaagcgagttcgtgtacggcgactacaaggtgtacgacgtgcggaagatgatcgccaagagcgagcaggaaatcggcaaggctaccgccaagtacttcttctacagcaacatcatgaactttttcaagaccgagattaccctggccaacggcgagatccggaagcggcctctgatcgagacaaacggcgaaaccggggagatcgtgtgggataagggccgggattttgccaccgtgcggaaagtgctgagcatgccccaagtgaatatcgtgaaaaagaccgaggtgcagacaggcggcttcagcaaagagtctatcctgcccaagaggaacagcgataagctgatcgccagaaagaaggactgggaccctaagaagtacggcggcttcgacagccccaccgtggcctattctgtgctggtggtggccaaagtggaaaagggcaagtccaagaaactgaagagtgtgaaagagctgctggggatcaccatcatggaaagaagcagcttcgagaagaatcccatcgactttctggaagccaagggctacaaagaagtgaaaaaggacctgatcatcaagctgcctaagtactccctgttcgagctggaaaacggccggaagagaatgctggcctctgccggcgaactgcagaagggaaacgaactggccctgccctccaaatatgtgaacttcctgtacctggccagccactatgagaagctgaagggctcccccgaggataatgagcagaaacagctgtttgtggaacagcacaagcactacctggacgagatcatcgagcagatcagcgagttctccaagagagtgatcctggccgacgctaatctggacaaagtgctgtccgcctacaacaagcaccgggataagcccatcagagagcaggccgagaatatcatccacctgtttaccctgaccaatctgggagcccctgccgccttcaagtactttgacaccaccatcgaccggaagaggtacaccagcaccaaagaggtgctggacgccaccctgatccaccagagcatcaccggcctgtacgagacacggatcgacctgtctcagctgggaggcgacAAAAAAAAAAAAAAAAAAAAAAAAAAAAAAAAAAAAAAAAAAAAAAAAAacaaagcaccagtggtctagtggtagaatagtaccctgccacggtacagacccgggttcgattcccggctggtgcaGCGGGGGACCTGGCCGGTGAGttttagagctagaaatagcaagttaaaataaggctagtccgttatcaacttgaaaaagtggcaccgagtcggtgcAacaaagcaccagtggtctagtggtagaatagtaccctgccacggtacagacccgggttcgattcccggctggtgcaATTTGGGCATGGTGGTTCAGGttttagagctagaaatagcaagttaaaataaggctagtccgttatcaacttgaaaaagtggcaccgagtcggtgcAacaaagcaccagtggtctagtggtagaatagtaccctgccacggtacagacccgggttcgattcccggctggtgcaGTGGAAAAGACAGAACCCGAGttttagagctagaaatagcaagttaaaataaggctagtccgttatcaacttgaaaaagtggcaccgagtcggtgcAacaaagcaccagtggtctagtggtagaatagtaccctgccacggtacagacccgggttcgattcccggctggtgcaGTGAAATTAACCAGCTGCAGGttttagagctagaaatagcaagttaaaataaggctagtccgttatcaacttgaaaaagtggcaccgagtcggtgcAacaaagcaccagtggtctagtggtagaatagtaccctgccacggtacagacccgggttcgattcccggctggtgcagatcgttcaaacatttggcaataaagtttcttaagattgaatcctgttgccggtcttgcgatgattatcatataatttctgttgaattacgttaagcatgtaataattaacatgtaatgcatgacgttatttatgagatgggtttttatgattagagtcccgcaattatacatttaatacgcgatagaaaacaaaatatagcgcgcaaactaggataaattatcgcgcgcggtgtcatctatgttactagatc

STU-Csy4(GmUBQ-Csy4-P2A-Cas9-Poly A- Csy4 cleavage site-sgRNA1-Csy4 cleavage site-sgRNA2-Csy4 cleavage site-sgRNA3-Csy4 cleavage site-sgRNA4-NOS)

tccttaagttgcagcatttaacacatctcctcattagagaaaaaaattcttccctaaacgatagtaaaaacatctaataagaaataagaaagaaaaattaggaaaaagaaaagttcattaaaaaaatcttttggattatttttaaaaaaatatctaaatattttttaaatgaataattttatataaactgtaactaaaagtatacaagtaatgtatgttaacaaaatacttgaaaaatctactgaaaatatatcttacaaagtgaaattaaataagaaagaatttagtggaataattatgattttatttaaaaaataattattaaagatttttttgctccataataagaaaacttttcaattattcttttctggtccataataaaaaaaatctagcatgacagcttttccatagatttttaataatgtaaaagcagccgacttcaggcaatggatagtggggcccgtatcaacttcggacgctccacttgcaacggggtgggcccaatataacaacgacgtcgtaacagataaagcgGagcttgaaggtgcatgtgactccgtcaagattacgaaaccgccaactaccacgcaaattgcaattctcaatttcctagaaggactctccgaaaatgcatccaataccaaatattacccgtgtcataggcaccaagtgacaccatacatgaacacgcgtcacaatatgactggagaagggttccacaccttatgctataaaacgccccacacccctcctccttccttcgcagttcaattccaatatattccattctctctgtgtatttccctacctctcccttcaaggttagtcgatttcttctgtttttcttcttcgttctttccatgaattgtgtatgttctttgatcaatacgatgttgatttgattgtgttttgtttggtttcatcgatcttcaattttcataatcagattcagcttttattatctttacaacaacgtccttaatttgatgattctttaatcgtagatttgctctaattagagctttttcatgtcagatccctttacaacaagccttaattgttgattcattaatcgtagattagggcttttttcattgattacttcagatccgttaaacgtaaccatagatcagggctttttcatgaattacttcagatccgttaaacaacagccttattttttatacttctgtggtttttcaagaaattgttcagatccgttgacaaaaagccttattcgttgattctatatcgtttttcgagagatattgctcagatctgttagcaactgccttgtttgttgattctattgccgtggattagggttttttttcacgagattgcttcagatccgtacttaagattacgtaatggattttgattctgatttatctgtgattgttgactcgacagaatggatcattatcttgatattagacttagacctgatccagaatttccaccagctcaacttatgtctgttctttttggaaaacttcatcaagctcttgttgctcaaggaggagatagaattggagtttcttttcctgatcttgatgaatcaagatcaagacttggagaaagacttagaattcatgcttctgctgatgatcttagagctttgcttgctagaccttggcttgaaggacttagagatcatcttcaatttggagaaccagctgttgttccacatccaactccttatagacaagtttcaagagttcaagctaaatctaatccagaaagacttagaagaagacttatgagaagacatgatctttctgaagaagaagctagaaaaagaattcctgatactgttgctagagctttggatttgccttttgttacacttagatcacaatctactggacaacattttagactttttattagacatggaccacttcaagttactgctgaagaaggaggatttacttgttatggactttctaagggaggttttgttccttggtttGGATCTGGTGCTACTAACTTCTCACTTTTGAAGCAAGCAGGAGATGTTGAGGAAAATCCAGGTCCTATGgacaagaagtacagcatcggcctggacatcggcaccaactctgtgggctgggccgtgatcaccgacgagtacaaggtgcccagcaagaaattcaaggtgctgggcaacaccgaccggcacagcatcaagaagaacctgatcggagccctgctgttcgacagcggcgaaacagccgaggccacccggctgaagagaaccgccagaagaagatacaccagacggaagaaccggatctgctatctgcaagagatcttcagcaacgagatggccaaggtggacgacagcttcttccacagactggaagagtccttcctggtggaagaggataagaagcacgagcggcaccccatcttcggcaacatcgtggacgaggtggcctaccacgagaagtaccccaccatctaccacctgagaaagaaactggtggacagcaccgacaaggccgacctgcggctgatctatctggccctggcccacatgatcaagttccggggccacttcctgatcgagggcgacctgaaccccgacaacagcgacgtggacaagctgttcatccagctggtgcagacctacaaccagctgttcgaggaaaaccccatcaacgccagcggcgtggacgccaaggccatcctgtctgccagactgagcaagagcagacggctggaaaatctgatcgcccagctgcccggcgagaagaagaatggcctgttcggaaacctgattgccctgagcctgggcctgacccccaacttcaagagcaacttcgacctggccgaggatgccaaactgcagctgagcaaggacacctacgacgacgacctggacaacctgctggcccagatcggcgaccagtacgccgacctgtttctggccgccaagaacctgtccgacgccatcctgctgagcgacatcctgagagtgaacaccgagatcaccaaggcccccctgagcgcctctatgatcaagagatacgacgagcaccaccaggacctgaccctgctgaaagctctcgtgcggcagcagctgcctgagaagtacaaagagattttcttcgaccagagcaagaacggctacgccggctacattgacggcggagccagccaggaagagttctacaagttcatcaagcccatcctggaaaagatggacggcaccgaggaactgctcgtgaagctgaacagagaggacctgctgcggaagcagcggaccttcgacaacggcagcatcccccaccagatccacctgggagagctgcacgccattctgcggcggcaggaagatttttacccattcctgaaggacaaccgggaaaagatcgagaagatcctgaccttccgcatcccctactacgtgggccctctggccaggggaaacagcagattcgcctggatgaccagaaagagcgaggaaaccatcaccccctggaacttcgaggaagtggtggacaagggcgcttccgcccagagcttcatcgagcggatgaccaacttcgataagaacctgcccaacgagaaggtgctgcccaagcacagcctgctgtacgagtacttcaccgtgtataacgagctgaccaaagtgaaatacgtgaccgagggaatgagaaagcccgccttcctgagcggcgagcagaaaaaggccatcgtggacctgctgttcaagaccaaccggaaagtgaccgtgaagcagctgaaagaggactacttcaagaaaatcgagtgcttcgactccgtggaaatctccggcgtggaagatcggttcaacgcctccctgggcacataccacgatctgctgaaaattatcaaggacaaggacttcctggacaatgaggaaaacgaggacattctggaagatatcgtgctgaccctgacactgtttgaggacagagagatgatcgaggaacggctgaaaacctatgcccacctgttcgacgacaaagtgatgaagcagctgaagcggcggagatacaccggctggggcaggctgagccggaagctgatcaacggcatccgggacaagcagtccggcaagacaatcctggatttcctgaagtccgacggcttcgccaacagaaacttcatgcagctgatccacgacgacagcctgacctttaaagaggacatccagaaagcccaggtgtccggccagggcgatagcctgcacgagcacattgccaatctggccggcagccccgccattaagaagggcatcctgcagacagtgaaggtggtggacgagctcgtgaaagtgatgggccggcacaagcccgagaacatcgtgatcgaaatggccagagagaaccagaccacccagaagggacagaagaacagccgcgagagaatgaagcggatcgaagagggcatcaaagagctgggcagccagatcctgaaagaacaccccgtggaaaacacccagctgcagaacgagaagctgtacctgtactacctgcagaatgggcgggatatgtacgtggaccaggaactggacatcaaccggctgtccgactacgatgtggaccatatcgtgcctcagagctttctgaaggacgactccatcgacaacaaggtgctgaccagaagcgacaagaaccggggcaagagcgacaacgtgccctccgaagaggtcgtgaagaagatgaagaactactggcggcagctgctgaacgccaagctgattacccagagaaagttcgacaatctgaccaaggccgagagaggcggcctgagcgaactggataaggccggcttcatcaagagacagctggtggaaacccggcagatcacaaagcacgtggcacagatcctggactcccggatgaacactaagtacgacgagaatgacaagctgatccgggaagtgaaagtgatcaccctgaagtccaagctggtgtccgatttccggaaggatttccagttttacaaagtgcgcgagatcaacaactaccaccacgcccacgacgcctacctgaacgccgtcgtgggaaccgccctgatcaaaaagtaccctaagctggaaagcgagttcgtgtacggcgactacaaggtgtacgacgtgcggaagatgatcgccaagagcgagcaggaaatcggcaaggctaccgccaagtacttcttctacagcaacatcatgaactttttcaagaccgagattaccctggccaacggcgagatccggaagcggcctctgatcgagacaaacggcgaaaccggggagatcgtgtgggataagggccgggattttgccaccgtgcggaaagtgctgagcatgccccaagtgaatatcgtgaaaaagaccgaggtgcagacaggcggcttcagcaaagagtctatcctgcccaagaggaacagcgataagctgatcgccagaaagaaggactgggaccctaagaagtacggcggcttcgacagccccaccgtggcctattctgtgctggtggtggccaaagtggaaaagggcaagtccaagaaactgaagagtgtgaaagagctgctggggatcaccatcatggaaagaagcagcttcgagaagaatcccatcgactttctggaagccaagggctacaaagaagtgaaaaaggacctgatcatcaagctgcctaagtactccctgttcgagctggaaaacggccggaagagaatgctggcctctgccggcgaactgcagaagggaaacgaactggccctgccctccaaatatgtgaacttcctgtacctggccagccactatgagaagctgaagggctcccccgaggataatgagcagaaacagctgtttgtggaacagcacaagcactacctggacgagatcatcgagcagatcagcgagttctccaagagagtgatcctggccgacgctaatctggacaaagtgctgtccgcctacaacaagcaccgggataagcccatcagagagcaggccgagaatatcatccacctgtttaccctgaccaatctgggagcccctgccgccttcaagtactttgacaccaccatcgaccggaagaggtacaccagcaccaaagaggtgctggacgccaccctgatccaccagagcatcaccggcctgtacgagacacggatcgacctgtctcagctgggaggcgacGGATCTGGTGCTACTAACTTCTCACTTTTGAAGCAAGCAGGAGATGTTGAGGAAAATCCAGGTCCTctcgagAAAAAAAAAAAAAAAAAAAAAAAAAAAAAAAAAAAAAAAAAAAAAAAAGTTCACTGCCGTATAGGCAGGCGGGGGACCTGGCCGGTGAgttttagagctagaaatagcaagttaaaataaggctagtccgttatcaacttgaaaaagtggcaccgagtcggtgcGTTCACTGCCGTATAGGCAGATTTGGGCATGGTGGTTCAGgttttagagctagaaatagcaagttaaaataaggctagtccgttatcaacttgaaaaagtggcaccgagtcggtgcGTTCACTGCCGTATAGGCAGGTGGAAAAGACAGAACCCGAgttttagagctagaaatagcaagttaaaataaggctagtccgttatcaacttgaaaaagtggcaccgagtcggtgcGTTCACTGCCGTATAGGCAGGTGAAATTAACCAGCTGCAGgttttagagctagaaatagcaagttaaaataaggctagtccgttatcaacttgaaaaagtggcaccgagtcggtgcGTTCACTGCCGTATAGGCAGgatcgttcaaacatttggcaataaagtttcttaagattgaatcctgttgccggtcttgcgatgattatcatataatttctgttgaattacgttaagcatgtaataattaacatgtaatgcatgacgttatttatgagatgggtttttatgattagagtcccgcaattatacatttaatacgcgatagaaaacaaaatatagcgcgcaaactaggataaattatcgcgcgcggtgtcatctatgttactagatc
